# Finding a Depression App: A Review and Content Analysis of the Depression App Marketplace

**DOI:** 10.2196/mhealth.3713

**Published:** 2015-02-16

**Authors:** Nelson Shen, Michael-Jane Levitan, Andrew Johnson, Jacqueline Lorene Bender, Michelle Hamilton-Page, Alejandro (Alex) R Jadad, David Wiljer

**Affiliations:** ^1^Centre for Addictions and Mental Health (CAMH)CAMH EducationToronto, ONCanada; ^2^Institute of Health Policy, Management and EvaluationUniversity of TorontoToronto, ONCanada; ^3^Centre for Global eHealth InnovationToronto General HospitalToronto, ONCanada; ^4^ELLICSR Health, Wellness and Cancer Survivorship CentreToronto General HospitalToronto, ONCanada; ^5^Faculty of MedicineDepartment of PsychiatryUniversity of TorontoToronto, ONCanada

**Keywords:** mobile apps, depression, health information, consumer, mental health

## Abstract

**Background:**

Depression is highly prevalent and causes considerable suffering and disease burden despite the existence of wide-ranging treatment options. Mobile phone apps offer the potential to help close this treatment gap by confronting key barriers to accessing support for depression.

**Objectives:**

Our goal was to identify and characterize the different types of mobile phone depression apps available in the marketplace.

**Methods:**

A search for depression apps was conducted on the app stores of the five major mobile phone platforms: Android, iPhone, BlackBerry, Nokia, and Windows. Apps were included if they focused on depression and were available to people who self-identify as having depression. Data were extracted from the app descriptions found in the app stores.

**Results:**

Of the 1054 apps identified by the search strategy, nearly one-quarter (23.0%, 243/1054) unique depression apps met the inclusion criteria. Over one-quarter (27.7%, 210/758) of the excluded apps failed to mention depression in the title or description. Two-thirds of the apps had as their main purpose providing therapeutic treatment (33.7%, 82/243) or psychoeducation (32.1%, 78/243). The other main purpose categories were medical assessment (16.9%, 41/243), symptom management (8.2%, 20/243), and supportive resources (1.6%, 4/243). A majority of the apps failed to sufficiently describe their organizational affiliation (65.0%, 158/243) and content source (61.7%, 150/243). There was a significant relationship (*χ* ^2^_5_=50.5, *P*<.001) between the main purpose of the app and the reporting of content source, with most medical assessment apps reporting their content source (80.5%, 33/41). A fifth of the apps featured an e-book (20.6%, 50/243), audio therapy (16.9%, 41/243), or screening (16.9%, 41/243) function. Most apps had a dynamic user interface (72.4%, 176/243) and used text as the main type of media (51.9%, 126/243), and over a third (14.4%, 35/243) incorporated more than one form of media.

**Conclusion:**

Without guidance, finding an appropriate depression app may be challenging, as the search results yielded non-depression–specific apps to depression apps at a 3:1 ratio. Inadequate reporting of organization affiliation and content source increases the difficulty of assessing the credibility and reliability of the app. While certification and vetting initiatives are underway, this study demonstrates the need for standardized reporting in app stores to help consumers select appropriate tools, particularly among those classified as medical devices.

## Introduction

Depression is a serious, common, and recurring disorder linked to diminished functioning, quality of life, medical morbidity, and mortality [1]. There has been a 37.5% increase in health life years lost to depression over the past two decades [2]. Depression was the third-leading cause of global burden of disease in 2004 and the leading cause of burden of disease in high- and middle-income countries. It is projected to be the leading cause globally in 2030 [3]. While effective treatments for depression are available, they are underused. Barriers to treatment include geography, socioeconomic status, system capacity, treatment costs (direct and indirect), low mental health literacy, cultural beliefs, and stigma [4,5]. A 2010 study found that 75% of primary care patients with depression in urban areas could identify more than one structural, psychological, cultural, or emotional barrier to accessing behavioral treatments. The rate was substantially higher in rural areas [6].

Information and communication technologies (ICTs) hold tremendous promise to expand the reach of quality mental health care [7] and close the treatment gap for depression. A meta-analysis [8] examining the effectiveness and acceptability of computer-based therapy for anxiety and depressive disorders found that computer-based therapy showed superiority in outcome over the control groups with substantial effect sizes. The study also found that adherence and satisfaction were good, suggesting acceptability. These findings were echoed in other meta-analysis studies of computer-based treatments for depression [9,10]. With the ever-increasing ubiquity and sophistication of ICTs, namely the evolution to mobile devices (ie, smartphones, tablets, and phone tablets or “phablets”), there is potential to further expand the reach of mental health treatment through mobile health (or mHealth). The emergence of a commercial marketplace of software for mobile devices (or apps) has given users the ability to personalize their devices to cater to their health and informational needs by purchasing or downloading apps at their convenience [11]. These apps can help support a variety of useful tasks such as self-assessment, symptom monitoring, psychoeducation, psychological therapy, and psychotherapy skills training [12].

Many consider apps as an opportunity to increase patient access to evidence-based mental health (and addictions) treatments [13-17]; however, many apps fail to incorporate evidence-based practices, health behavior theory, or clinical expertise [17-19] into the design of the app. For instance, smoking cessation apps are found to have low adherence to evidence-based practices [20,21] and insufficiently incorporate behavioral theory [22]. A study on addiction recovery apps found that only six of the 52 app developers had clinical experience or used academic or clinical advisors in the development of apps; additionally, none of the app store descriptions mention any evaluation of the apps [23]. The lack of reported evaluations is also seen in scientific literature, as the current body of evidence is marginal in comparison to the number of mental health apps available. In 2013, there were only 32 published articles on depression apps in comparison to the 1536 available in the marketplace [24]. A 2013 systematic review [14] found only four studies (3 randomized controlled trials and 1 pre-post) evaluating three different depression apps. Two apps demonstrated a significant reduction in depression [25,26]; however, none of the apps were publicly available at the time of that review.

The discrepancy between availability and evaluation is problematic because many of these products will continue to be marketed with unfounded claims of health improvement to attract health consumers [27-29]. To better understand what types of apps are offered to those seeking support for depression, this study aimed to identify the mHealth offerings in the mobile app marketplace and characterize the information provided to health consumers in the app store descriptions. This study asked the following research questions: (1) What mobile apps are available for people in treatment for depression, as well as for their families, including informal caregivers? (2) What are the commercial characteristics of depression apps? (3) What are the main purposes of depression apps? and (4) How do depression apps claim to support users in the store description?

##  Methods

### Overview

We used a systematic review and content analysis approach based on a study by Bender et al [30] to guide the collection and characterization of available depression apps. The review was carried out on the five major app stores: Apple (iTunes), Android (Google Play), BlackBerry (AppWorld), Nokia/Symbian (Ovi), and Windows Mobile (Marketplace). On March 5, 2013, we entered the keyword “depression” into the search field on each of the four marketplace websites. The Apple apps were accessed through the iTunes interface using the same search term. The search term was applied across all store categories in the five instances. The two reviewers (MJL and NS) recorded the links and the titles of apps found in the search yield. Based on their availability, one reviewer (NS) compiled apps found in iTunes and the other (MJL) focused on the remaining app stores. For the eligibility assessment of the apps, the entire inventory was split into two equal samples for independent review.

### Selection Criteria

Apps were organized as either “potentially relevant” or “not relevant” based on the app title, store description, and available screenshots. Apps were categorized as “potentially relevant” and included in the final analysis if they met three criteria: (1) the term “depression” was in the title or store description, (2) the app targeted health consumers (ie, those who self-identify as needing support for depression, including family or caregivers), rather than health care professionals, and (3) the app had an English-language interface or English translation (if in another language).

Apps were excluded from the study if they did not provide sufficient information, did not have a clear focus on depression, used the term depression in an unrelated context (eg, the Great Depression), used the term depression as a keyword in a list of unrelated items or as background information, and were duplicates appearing in multiple markets or for other devices (ie, optimized for tablets). The duplicate that provided the most information for data extraction was retained based on the following hierarchy (most to least information): Google Play, iTunes, AppWorld, Ovi, and Marketplace.

After independent screening for relevance, the 2 reviewers exchanged a random selection of 5% (104 apps) of their search yields to verify eligibility. Interrater reliability (IRR) of the random samples, as determined by Cohen’s kappa (kappa=.77, *P*<.001), was statistically significant. According to Landis and Koch’s guidelines [31], the score indicated that there was a “substantial agreement” between the 2 reviewers. Because the IRR exceeded the pre-determined minimum kappa threshold of .7, independent reviews of the whole sample were not required. Disagreements found in the exchanged sample were resolved by consensus.

### Data Extraction and Coding

Information was extracted from the store descriptions of the apps for the following variables: commercial information (ie, year of release/update, cost, developer name, audience, downloads), organizational affiliation, content source, main purpose, user interface, media type, and popularity (ie, rating, number of raters, number of comments). The 2 reviewers (MJL and NS) collectively and iteratively developed a preliminary coding scheme by analyzing the content of 20.5% (108/528) of randomly selected “potentially relevant” apps. The coding for the main purpose variable used the Luxton et al [17] classification of mental health app (ie, self-assessment, symptom monitoring, psychoeducation, psychological therapy, psychotherapeutic skills training) as the foundation for development. An IRR test of 20.4% (22/108) of this pilot sample was conducted to evaluate understanding and application of the codes. The results were all significant (*P*<.001), yielding “almost perfect” agreement for exclusion (kappa=1.00), affiliation (kappa=.91), content source (kappa=1.00), and user interface (kappa=.91). There was “substantial agreement” for main purpose (kappa=.77) and “moderate agreement” for media type (kappa=.49) [31]. The discrepancies in coding for the multimedia variable were discussed, and problem areas were identified and resolved. The final coding scheme is outlined in Table 1.

The remaining sample was divided for data extraction based on odd and even numbering to ensure that the reviewers had equal proportions of apps from each marketplace. After independent review, 20% (combined 41 apps) of each reviewer’s sample was randomly selected, exchanged, and coded to assess IRR. The results were all significant (*P*<.001) with “almost perfect agreement” for affiliation (kappa=.89) and main purpose (kappa=.83), and “substantial agreement” for user interface (kappa=.74). There was also “substantial agreement” for media type (kappa=.68); however, the low kappa (kappa<.70) required the reviewers to examine and understand the discrepancies in coding and correct the coding within each of their respective samples. This process was also applied to content source (kappa=.53). Flagged apps were collectively reviewed for inclusion and then coded. Because the exclusion criteria became more nuanced during this process, apps that were labeled not relevant were also collectively reviewed and coded if they were considered relevant.

**Table 1:** Final codebook for content analysis.

Variable	Code	Description
Organizational affiliation	UNI	UNIVERSITY: Produced in affiliation with a university or other academic institution
	MEDC	MEDICAL CENTER: Produced in affiliation with a medical institution
	GOVT	GOVERNMENT: Produced in affiliation with a government institution
	INST	INSTITUTION: An explicit association (ie, foundation, center, NGO, church)
	OTHER	OTHER: There is a clear but unclassifiable affiliation (eg, LLC, LLP, Inc.), not .com
	INSUFF	INSUFFICIENT: The affiliation cannot be confirmed by available info
Content source	EXP	EXPERT: Developed by/with an accredited medical professional (eg, Dr., LCSW)
	EXT	EXTERNAL SOURCE: From specific external source (eg, BDI, DSM, Bible) but not “based on” or inspired by a theory/practice (eg, cognitive behavioral therapy)
	LAY	LAYPERSON: Source identified but no credential mentioned. Non-medical expertise clearly indicated by detailed bio or qualifier (eg, years of experience)
	PLE	PERSON LIVED EXPERIENCE: Indication that app is developed by people with lived experience
	INSUFF	INSUFFICIENT: No direct information provided about origin of intervention
Audience	ADULT	ADULT: Adult or high maturity, age 18+
	YADULT	YOUNG ADULT: Medium maturity, age 12+
	YOUTH	YOUTH: Low maturity, age 9+
	ALL	ALL: “Everyone,” age 4+, “general,” no rating
Main purpose	PE	PSYCHOEDUCATION: Educational material that includes books or guides, news or journal articles, commentaries/opinions, tips, and lessons
	MA	MEDICAL ASSESSMENT: Allows users to screen, diagnose, assess risk, determine treatment
	SM	SYMPTOM MANAGEMENT: Allows users to track symptoms – only for mood diaries
	SR	SUPPORTIVE RESOURCES: Provides referrals for help or connects users with support. May include the use of forums
	TT	THERAPEUTIC TREATMENT: Provides therapy and includes functions that support relaxation (eg, hypnosis, binaural beats); meditation, spiritual faith-based solutions; holistic therapy (eg, diet, exercise, nutrition, lifestyle, cannabis); and positive affirmation
	MULTI	MULTIPLE PURPOSES: Use only if indistinguishable overlap of categories
User interface	INFO	INFORMATION ONLY: Static user interface that provides minimal interaction (eg, e-book). The only interactions available are for settings or navigation
	TOOL	TOOL: Dynamic user interface that provides an interactive component to app (ie, games, social media consultation) or allows users to input data
Media type	AUD	AUDIO: Audio only (with supporting background images/text)
	TXT	TEXT ONLY: Text only (with supporting background images) – eg, e-book
	PIC	PICTORIAL: Pictures only (eg, wallpaper)
	VID	VIDEO: Video only
	VIS	VISUAL: Animations or graphics or charts (ie, no audio or video)
	MULTI	MULTIMEDIA: Used more than one of the categories above
	INSUFF	INSUFFICIENT: Not enough information to determine types of media used

### Data Analysis

Cohen’s kappa and descriptive statistics were computed using SPSS version 20. Chi-square tests of independence examined the relationship between the variables data source, user interface and multimedia, and the main purpose of the app. Statistical significance was set at *P*<.05. The option to collapse the values within a variable to fulfill the expected cell frequency assumptions of chi-square tests was explored if the research team viewed it as a logical transformation.

## Results

### General Characteristics

**Figure 1:**
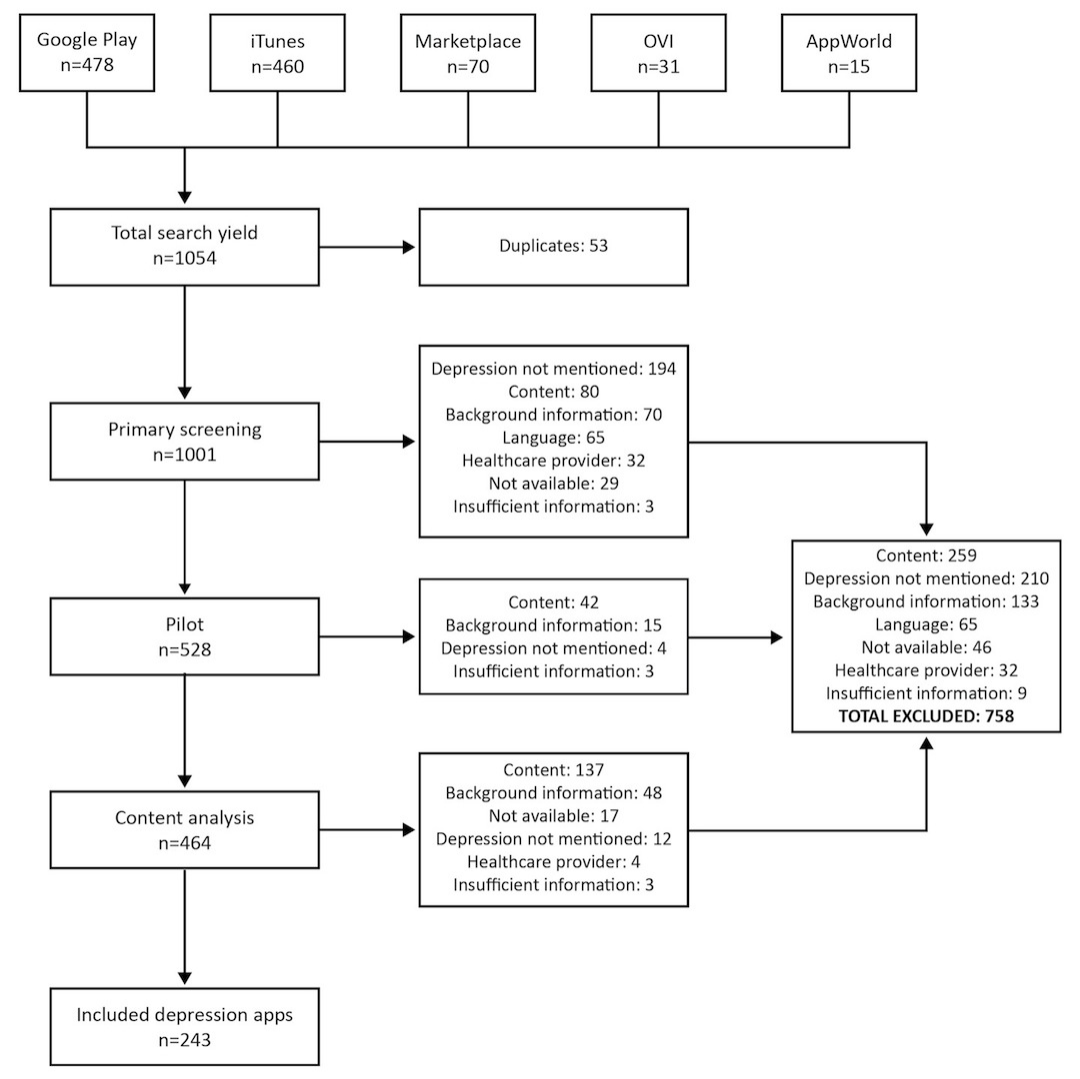
Flow diagram illustrating the exclusion of apps at various stages of the study.

The initial search yielded 1054 apps, of which 53 were excluded as duplicates (31 were available in two stores, eight in three stores, two in four stores, and one in all stores). Of the remaining apps, 243 met the inclusion criteria. Figure 1 shows the exclusion of apps at the various stages of the study. See Multimedia Appendix 1 for a list of the included apps.

Windows (4.5%, 11/243), Nokia (2.5%, 6/243), and BlackBerry (2.5%, 6/243) accounted for less than 10% of the included sample, as the majority of apps were from the Google (53.5%, 130/243) and Apple (37.0%, 90/243) marketplaces. The apps spanned 32 different store categories, with 79.9% (194/243) of the apps found under four categories: health and fitness (41.2%, 100/243), medical (17.3%, 42/243), lifestyle (14.4%, 35/243), and books (7.0%, 17/243). Six (2.5%, 6/243) apps had no categorization. The average price for paid apps (152/243; 62.6%) was CAN $3.15 and ranged from $0.99 to $15.99. The majority of paid apps (73.7%, 112/152) were sold for less than $4.99, with the mode price of $0.99 (18.9%, 46/243).

Only the release date was provided by the iTunes store, whereas Google Play, BlackBerry, and Windows provided dates of the last app update. Nokia did not provide this information. The earliest date reported by the app stores was 2009 (3.7%, 9/243). Two-thirds (66.0%, 156/237) of the apps were released or updated in 2012 (36.2%, 88/243) and the first quarter of 2013 (28.0%; 68/243). Google Play was the only market that reported the number of installs (ie, downloaded and installed on an Android mobile device) and was reported in ranges; 40 apps (30.8%, 40/130) were installed less than 50 times. The most frequent ranges of installation were 100-500 and 1000-5000, each registering 16.9% (22/130) of the sample. One app (0.4%, 1/243) was installed in the 1 million to 5 million range, and four apps fell into the 100,000 to 500,000 range.

### Developers and Affiliations

There were 190 developers in the sample, with 35 accounting for multiple apps. Of this group, 27 developers created two apps, three developed three apps, and four developed four apps. The top developer, MOZ, created nine apps. Only 5.3% (10/190) of the developers were either medical centers (1.0%, 2/190), universities (1.0%, 2/190), and institutions (3.2%, 6/190). A total of 56 developers indicated that they were a commercial developer (eg, LLC, LLP, Inc.), while 124 developers did not provide sufficient information about their affiliation.

### Depression Apps Ratings

Of the 113 rated apps (46.5%, 113/243), there was an average of 37.2 raters (95% CI 21.6-52.81) per app. One app had 583 raters. The average rating (out of five stars) was 3.5 stars (95% CI 3.3-3.7). There was an average of 5.9 comments per rated app (95% CI 4.2-7.7), with a range from zero to 56 comments.

### Overall Picture of Depression Apps

Over 80% of the apps had the main purpose of providing therapeutic treatment (33.7%, 82/243), psychoeducation (32.1%, 78/243), or medical assessment (16.9%, 21/243). Apps with multiple purposes accounted for 7.4% (18/243) of the sample. Only 38.3% (93/243) of the apps reported the content source in sufficient detail and mainly cited an external (17.7%, 42/243) or expert (14.0%, 30/243) source. The majority (72.4%, 176/243) featured a dynamic user interface. Over half of the apps were text-only (51.9%, 126/243), while 14.4% (35/243) used multiple forms of media. Table 2 summarizes the distribution of apps across the different variables.

The chi-square tests of independence yielded significant results (*P*<.001); however, the expected cell count assumption was violated in all cases. Two variables, affiliation and content source, were collapsed into binary variables. The chi-square analysis for affiliation (ie, sufficiently or insufficiently reported) and main purpose showed that there was no relationship between the two variables (χ^2^_5_=8.8, *P*=.12). The content source variable (ie, sufficiently or insufficiently reported) showed a significant (χ^2^_5_=50.5, *P*<.01) association between the main purpose of the app and the reporting of the source. An ad hoc analysis was conducted between media type and user interface, which yielded a significant relationship between the two variables (χ^2^_4_=46.3, *P*<.01).

**Table 2:** Distribution of depression apps by variable and main purpose.

Variable and Value			Main purpose, n (%)^a^						
			TT	PE	MA	SM	SR	MP	Total
Overall^b^			82 (33.7)	78 (32.1)	41 (16.9)	20 (8.2)	4 (1.6)	18 (7.4)	243
**Affiliation** ^c^									
	**Reported** ^b^ **85 (35.0)**								
		Institution	1 (1.2)	2 (2.6)		1 (5.0)	3 (75.0)		7 (2.9)
		Academic			1 (2.4)		1 (25.0)		2 (0.8)
		Medical center	1 (1.2)		1 (2.4)				2 (0.8)
		Other	27 (32.9)	21 (26.9)	14 (34.1)	6 (30.0)		6 (33.3)	74 (30.5)
	Insufficient information		53 (64.6)	55 (70.5)	25 (61.0)	13 (65.0)		12 (66.6)	158 (65.0)
**Content source** ^c^									
	**Reported 93 (38.3)**								
		External	8 (9.8)	6 (7.7)	21 (26.9)	1 (5.0)	1 (25.0)	5 (27.8)	42 (17.3)
		Expert	3 (3.7)	10 (12.8)	11 (26.8)			6 (33.3)	30 (12.3)
		Patient lived experience		7 (9.0)	1 (2.4)	2 (10.0)		1 (5.6)	11 (4.5)
		Layperson	9 (11.0)	1 (1.3)					10 (4.1)
	Insufficient information		62 (75.6)	54 (69.2)	8 (19.5)	17 (85.0)	3 (75.0)	6 (33.3)	150 (61.7)
**User interface**									
	Tool (dynamic)		75 (91.5)	18 (23.1)	41 (100.0)	20 (100.0)	4 (100.0)	18 (100.0)	176 (72.4)
	Information only (static)		7 (8.5)	60 (76.9)					67 (27.6)
**Media type** ^d^									
	Text only		17 (20.7)	61 (78.2)	35 (85.4)	4 (20.0)	2 (50.0)	7 38.9)	126 (51.9)
	Audio only		36 (43.9)	3 (3.8)					39 (16.0)
	Multimedia		16 (19.5)	9 (11.5)	1 (2.4)	3 (15.0)	2 (50.0)	4 (22.2)	35 (14.4)
	Visual		8 (9.8)	4 (5.1)	4 (9.8)	11 (55.0)		7 (38.9)	34 (14.0)
	Pictorial		5 (6.1)			1 (5.0)			6 (2.5)
	Insufficient information			1 (1.3)	1 (2.4)	1 (5.0)			3 (1.2)

^a^Calculated as percentage within main purpose category; TT=therapeutic treatment, PE=psychoeducation, MA=medical assessment, SM=symptom management, SR=supportive resources, MP=multiple purposes.

^b^Total was calculated as percentage within the whole sample (N=243).

^c^The denoted variables were collapsed into binary categories for chi-square analysis.

^d^None of the apps were video based.

### Characterization of Apps by Main Purpose

#### Therapeutic Treatment

Audio (44%, 36/82) was the most frequently used media for therapeutic treatment apps, which accounted for 92% (36/39) of audio apps found in the entire sample. Similarly, therapeutic treatment apps most frequently used multimedia, which represented 46% (16/35) of multimedia apps in the entire sample. Half (41/82) of the therapeutic treatment apps supported audio therapy in the form of hypnosis (n=14), brainwave entrainment (n=23), music therapy (n=3), or nature sounds (n=1). Five of the audio therapy apps included other types of media. One hypnosis app used visual media only. Nine of the 11 relaxation therapy apps reported layperson as the source, which accounts for 90% (9/10) of the layperson-sourced apps in the sample. Other types of therapy included spiritual/faith-based (n=10), entertainment (n=10), positive affirmation (n=7), behavior training (n=7), and light/visual (n=3). Two apps provided exercise-based therapy consisting of breathing techniques and yoga. One app focused on diet and one provided activity suggestions. There were ten apps that provided cognitive behavioral therapy and were classified under the multipurpose category.

#### Psychoeducation

The psychoeducation category of apps predominantly used a static (ie, read-only) interface (n=60) and represented 90.0% (60/67) of the static interface apps in the sample. The most frequently used media was the text-only category (n=61) and represented roughly half of all text-only apps (48.4%; 61/126) in the entire sample. Fifty psychoeducation apps were general e-books about depression, of which two were fiction and seven were reference manuals (ie, medication library), 12 apps provided tips or advice on how to overcome depression, and 11 apps provided education through learning modules or lessons. Five apps provided a collection of resources such as news and journal articles. The psychoeducation category had the greatest number of apps based on patient lived experience (n=7). Five of these were general e-books, one provided tips, and one provided lessons.

#### Medical Assessment

Of the medical assessment apps, 33 (81%; 33/41) reported the content source, which is the highest proportion and number of sourced apps within a main purpose category. External sources were reported 21 times and used 11 different questionnaires. The most frequently used questionnaire was the Patient Health Questionnaire (PHQ-9) [32], used in eight apps. The Beck Depression Inventory 2 [33], Geriatric Depression Scale [34], and M3 Questionnaire [35] were all used twice. The Automatic Thoughts Questionnaire [36], Center for Epidemiology Studies Depression Scale [37], Edinburgh Postnatal Depression Scale (EPDS) [38], Goldberg Depression Questionnaire [39], Quick Inventory of Depressive Symptomology Questionnaire [40], and Zung Self-Rating Depression Scale (SDS) [41] were each used once. The Psychological Tests App contained multiple depression questionnaires. The 11 expert-sourced apps did not provide a specific questionnaire but mentioned in the description that a medical professional (ie, physician or psychologist) developed the app or that the questionnaire was used in practice. One app contained a questionnaire based on patient lived experience. With the exception of five apps, all the apps were text-only.

#### Symptom Management

Only 15% (3/20) of symptom management apps reported the content source, the lowest proportion of all the main purpose categories. Over half of the symptom management apps used visual media (55%; 11/20). Nine apps allowed users to track their moods and eight tracked lifestyle factors (eg, mood, sleep, diet, medication, exercise). Two apps allowed users to keep a journal, and one app used a checklist system.

#### Supportive Resources

Half of the apps (50%; 2/4) were text-only, while the other half were multimedia. One app reported the content source and cited an external source. Two apps provided resources (online and offline) and references for help. The other two apps connected users to a community via online forums.

#### Multipurpose

**Figure 2:**
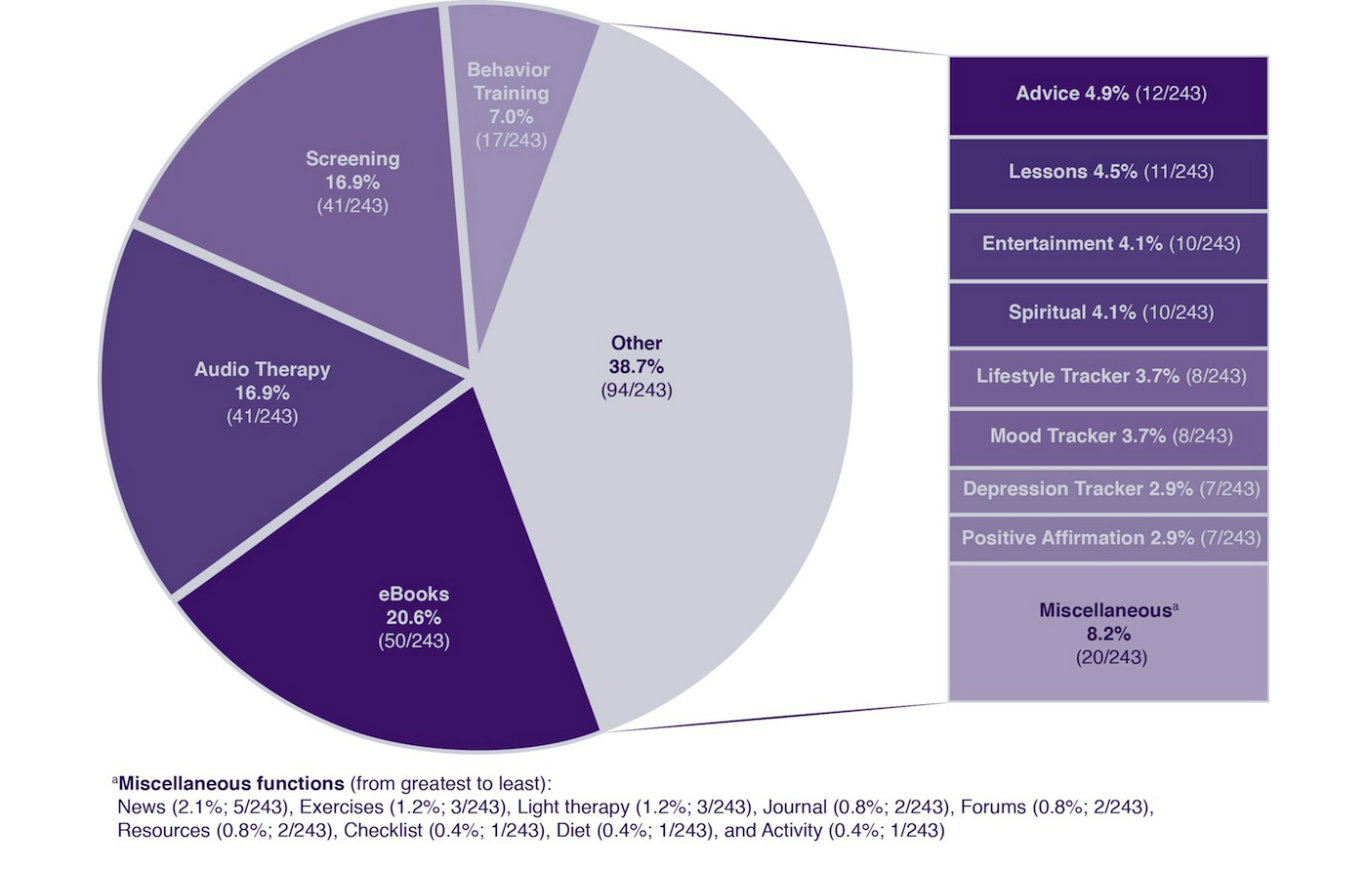
Distribution of depression apps by function.

Two-thirds (67%; 12/18) of the multipurpose apps reported the source, with almost all citing an expert (n=6) or external (n=5) source. All the apps used text (n=7) or visual (n=7) as the primary media. Four apps were multimedia, and 17 apps (94%; 17/18) used a combination of medical assessment and symptom management. Ten of these apps specifically focused on cognitive behavioral therapy (CBT), while seven used a questionnaire and allowed users to track depression over time. The questionnaires consisted of PHQ-9 (n=2) [32], EDPS (n=1) [38], and SDS (n=1) [41]. One app used a proprietary questionnaire (Treatment Depression Inventory). Two apps did not specify the questionnaire. One app provided therapeutic treatment through meditation exercises and also provided psychoeducation about the exercises and CBT. Figure 2 presents a summary and distribution of the different app functions.

## Discussion

### Principal Findings

**Figure 3:**
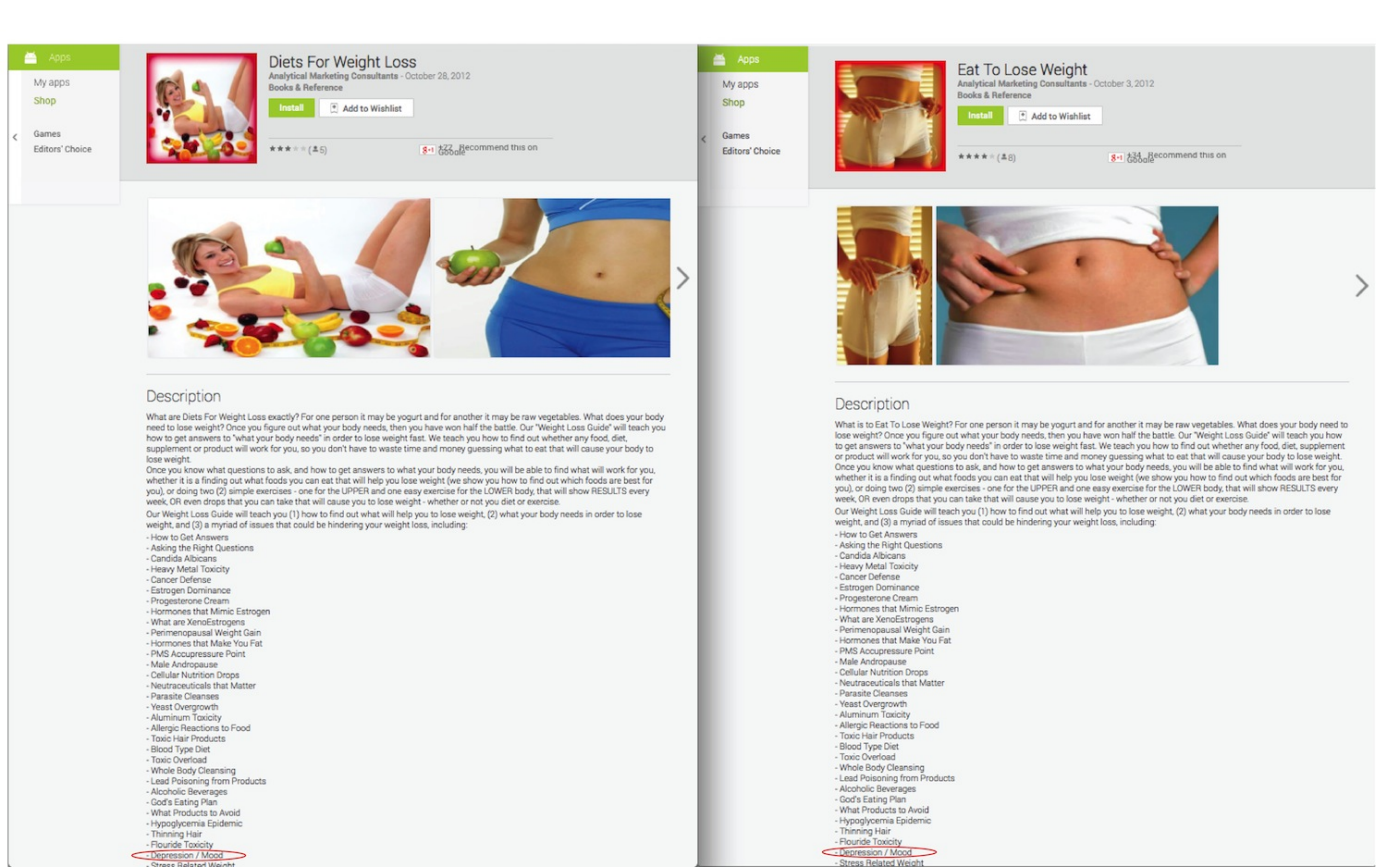
An example of white labeling where the apps have the same description but are labeled as different apps. The word depression (circled in red) is only one in a list of unrelated terms and is an example of how such lists allow non-depression apps to enter the search.

This review found that depression apps provided support on five different dimensions: therapeutic treatment, psychoeducation, medical assessment, and supportive resources. Through the iterative development of this typology and understanding of the available commercial information, the results provided some insights into the user experience of those seeking depression support through apps. Similar to a recent study by Martinez-Perez et al [24], this study found that depression app seekers need to filter through 400+ apps in either the Google Play or iTunes marketplace. In context of the one million app milestone announcements by both Google and Apple in 2013 [42,43], this number may suggest that the app marketplace has entered a phase of “overload” or “diseconomies of scale”, where the large quantity of apps available makes it difficult for users to find the right one [44,45]. The apps excluded from this study indicate that metadata may play a role in this phenomenon. Vendors may leverage the use of metadata or the keyword “depression” to increase exposure of their non-depression apps in the depression app search results. For example, one-fifth of the search yield made no mention of depression anywhere in the app title or store description. One-quarter of the search yield was excluded because the word depression was mentioned only in a “laundry list” of keywords in the app’s description, not in the title. Many of these apps were white-labeled (ie, essentially identical but marketed for different purposes or under different developer names) and were evident by the identical store descriptions (see Figure 3). White labeling was primarily observed for e-book and audio therapy apps. Last, although some apps made reference to depression, their main purpose was to address a different condition (eg, weight loss or acne apps may describe how being obese or having acne may lead to depression).

Of the apps included in the study, there were three times more text-only apps than any other media category; furthermore, almost all the text-only apps with static interfaces were found in the psychoeducation app category. The reviewers found that these apps, based on screenshots and descriptions, were rudimentary in function and minimal in design. The proliferation of these apps may be a result of the low barrier to entry into the marketplace in the form of prerequisite resources and skills, thereby allowing those with minimal programming skills and resources to develop and publish their own apps [46]. This finding could explain why only one-third of the 190 unique developers adequately described or indicated their affiliation and the proportionately low number of apps from formal institutions. Furthermore, only a third of the app store descriptions reported content sources. Many other app reviews [18-23,30,47-51] have also found that the app development process often failed to involve health care professionals or academics and to include content aligned with clinical guidelines or behavior change theories or techniques. The majority of these apps were categorized under the main purposes of psychoeducation and therapeutic treatment.

The lack of apps that incorporate authoritative sources remains problematic. It has been estimated that one in five of paid apps claim to treat or cure medical ailments [28]. Similar to the potential shortcomings of information found on the Internet, the information or therapies provided by apps may be incomplete or based on insufficient scientific evidence. This presents a potential health hazard for consumers who interpret this information incorrectly or try inappropriate treatments [52]. For example, reading about a disease may increase health anxiety, reinforce hypochondriasis, cause unnecessary concerns, or lead people to purchase harmful drugs or engage in risky health behaviors [53]. These harms, however, are often a cautionary claim, as most research on the utility of online health information has focused on the quality of information rather than its effects [54,55]. Only a few studies actually reported instances of harm [56]. This gap between evidence-based recommendations and app functionality continues to be a common theme across different health conditions [20,21,47,51,57-59]. Public attention has turned to these “snake oil” apps, prompted by a US Federal Trade Commission settlement involving two app developers who falsely cited a study from the *British Medical Journal of Dermatology* in their claims that the colored display screens featured in their apps could cure acne [60]. The proceedings were founded on the premise of false advertising rather than public safety [61]. This case has led to a call for the US Food and Drug Administration (FDA) to regulate mobile medical apps; however, there is debate about the appropriateness of this measure [62]. In September 2013, the FDA issued guidance for developers of apps that perform as medical devices, defined as apps that diagnose or treat disease whereby malfunctions can carry significant risks of harm [63].

Based on the app store categories used in this study, 42 apps were defined as medical; however, this category included apps that are considered innocuous, such as those that help patients organize their health information or look up information about treatments [64]. Perhaps these apps would be better suited for other categories, such as health and fitness, lifestyle, and books, where more than half of the included apps were found. Apps found in these non-medical categories are considered low risk as long as they do not provide specific treatments or treatment suggestions. They may provide benefits to the patient, such as those associated with using a mood tracker to maintain a symptom diary [65]. To help users navigate the app marketplace, Happtique (a subsidiary company of the Greater New York Hospital Association) developed standards for an app certification program in early 2013. Unfortunately, these efforts were brought to a halt when an audit found that 2 of the 19 Happtique-certified apps had privacy issues [66]. There are other initiatives to help curate apps, such as the iMedicalApps website; however, it is a tremendous task to benchmark. Policing the quality of apps is a near-impossible endeavor that is reminiscent of the early days of appraising online health information [67]. Deshpande and Jadad have found that past initiatives to assess the quality of online health information or tools had limited success and recommend that efforts be hedged towards an open, distributed, and collaborative approach similar to Wikipedia [68].

### Evaluation

The most common function of depression apps provides users with information about depression through an e-book modality. Despite the potential to translate books or bibliotherapeutic guides, only 13 of the 50 e-books cited a content source. The majority of these books were self-help guides, often with titles that claimed they would help users overcome depression. Examples include “Beat Depression”, “Defeat Depression”, and “Stomping Out Depression”. While these non-sourced books do pose the potential to distribute erroneous or biased information to people seeking help, the Google dataset shows that two-thirds of these apps are installed less than 100 times and indicates that users do exercise some discretion before purchasing or installing apps. Nettleton et al [69] suggested that users are able to make reasonable assessments of health information in the context of other health information seeking practices to complement their formal care. This behavior extends to mobile phone apps: one qualitative study found that the reputation and legitimacy of sources factor into the use of an app [70]. For example, an e-book app that cited the US National Institutes of Health was downloaded within the 10,000 installs range. While promising, this finding could be confounded by the application’s free status. The “Anxiety and Depression” and “Audio Book Anxiety and Depression” e-book apps, which were in the install ranges of 10,000 and 100,000, were also free. One study suggested that consumers exercise more caution when having to purchase apps than when downloading them for free due to the burden of price [71]. The same study also showed that ranking, customer ratings, and content size affect downloading when the app is free. Consumers depend more on their own information and experiences rather than on rankings or ratings when the app requires payment. They closely consider low ratings, including complaints, not mean score when they have to pay [71]. The relationship between price, affiliation, source, downloads, and satisfaction via ratings and comments could be a potential area to explore in future studies.

Medical assessment was the only app category with a high rate of reporting content source. All of these apps were screening tools that allowed users to self-diagnose for depression. There is an absence of published data investigating the impact of patient self-diagnosis using apps or the Internet; however, some studies have identified false positive assessments as a potential source of harm [53,72-74]. Despite this shortcoming, medical assessment apps could help to address some systemic barriers to diagnosing depression in primary care [75]. Depression is often under-detected in the health care system, and the practice of routine screening is a contentious and unresolved issue [76]. Medical assessment apps may help to bridge this gap by assisting individuals in identifying mental health issues, thereby providing the impetus to approach and engage their health care providers. Clarke and Yarborough described this effect as a lowering of threshold of entry-level mental health services so that it extends the reach of care to people who do not seek traditional treatment for depression [5].

Audio therapy apps may have a similar potential to that of medical assessment apps [77,78]. This study found that half of therapeutic treatment used audio therapy and is consistent with a recent report that found that 43% of therapeutic apps used audio for treatment [28]. The effectiveness of audio therapy, regardless of mode of delivery, is not fully understood and is often under scrutiny [47,79-81]. There are many gaps in knowledge regarding the psychological effects of brainwave entrainment and hypnosis on depression [82,83]. Systematic reviews [81] and meta-analysis [84] of existing research have found mixed results on the effectiveness of these types of interventions. A similar review of a hypnosis app found on iTunes reported that none of the 407 identified apps were tested for efficacy or were based on evidence [47]; however, the study did not discuss potential harms associated with using non-evidence-based, non-evaluated apps. The authors do caution against “self-described professional titles”, as certification could easily be purchased online. They also warn that certification does not mean that the individual was adequately trained.

The fourth most prevalent function of depression apps was offering behavior training or therapy, with most apps focusing on CBT. Internet-based CBT (ICBT) has shown to be an effective treatment for depression [85], with the magnitude of effects depending on level of support and content of the intervention [86]. ICBT is considered to be well suited for delivery through an app because it would offer users the convenience of recording and tracking their moods and context in real time, as well as accessing psychoeducational materials [87]. Two-thirds of the CBT apps identified in this study had multiple purposes, which often included tracking, screening, and providing psychoeducation. In practice, one study demonstrated the feasibility of app-based CBT in treating depression, with clinical improvement in the patients [26]. This app was captured in the sample and provided a very brief description mentioning the CBT program and its affiliation with a hospital; however, the raters felt it did not provide sufficient information about the intervention source. This shortcoming underscores the importance for app developers to follow a standardized reporting system to advertise the credibility of apps and to prevent empirically tested apps from going unnoticed. Similarly, it might be necessary to develop a framework that could protect both app developers and users from harm, particularly from liability associated with cases of preventable suicide.

### Limitations

While the development of regulations and certification standards for assessing the quality of apps is underway, this study used the information available in the app store description (ie, developer affiliation and content source) to understand how depression apps are advertised to health consumers seeking depression apps. The information provided about affiliation and content source was accepted prima facie based on the developed inclusion criteria. The high percentage of insufficient reporting of affiliation may be an overestimation, since the developer websites were not examined to corroborate their status. Similarly, the reported content sources were not further examined. It is acknowledged that the apps themselves may contain more information and that not downloading and testing the apps is a limitation of this study. The lack of physical testing mirrors the actual user experience when making the decision to download apps [48], where the information provided in the description may serve as an initial proxy measure for quality before downloading and trialing an app. It also underscores the need for a standardized app store description reporting system for vendors to refer or adhere to. With over 190 unique developers identified in our eligible sample and many more in the initial sample, consumers may not have the time to view all the developer websites to verify their affiliations. Requiring vendors to outline their affiliations, evidence base, or content source could provide potential users with enough contexts to assess the credibility of the app.

A second limitation lies in the possibility that many of the apps excluded from this study because they were not depression specific could potentially be useful for people with depression. ICBT apps are prime examples of potentially useful non-depression-specific apps. ICBT is regarded as a well-established treatment for depression, panic disorder, and social phobia, but it is also an option for 25 other clinical disorders. While ICBT apps could be the prototypical depression app [26,88], non-depression ICBT apps were excluded to maintain consistency in assessing the relevance of other apps that provided an intervention (eg, binaural beats [81], yoga [89], spirituality [90]) where a case could be made for their inclusion. To prevent confirmation biases from entering the sample, it was decided that the app was required to be specific to depression to be eligible.

This study represents a snapshot of depression apps found in Canadian app stores in March of 2013. This may be a limitation in three ways. First, the landscape of the depression market will have changed at the time of submission of this publication. Second, the findings from this study may not be representative of all the depression apps available on the global market because certain apps may be localized or licensed only to specific countries. The study by Martinez-Perez et al in Spain found over 1537 depression apps available on the five major platforms. In comparison, the current review yielded 1001 unique apps, with a large part of the discrepancy attributed to Google Play app count. Moreover, a sample of Android apps may be missing because this study was conducted just prior to the Amazon announcement [91] of expanding access to its Android app store outside of the United States to Canada and 200 other countries. A quick search of the Android app store using the search term “depression” yielded 123 apps. Because development standards vary from different app stores, future content analysis studies should consider including the Amazon marketplace to understand its contributions to the app marketplace. Last, frameworks such the Self-Certification Model for Mobile Medical Apps by Health on the Net Foundation (HON) [92] and App Synopsis [93] became available shortly after the data extraction phase concluded (mid-2013). These models provide some important parameters that were not covered in this study (eg, data requisition and management, advertising policy, justification of claims). However, this study demonstrates that most apps would fare poorly against the aforementioned standards and delineates the need for such reporting approaches to be disseminated to mHealth developers to bring the information presented to health consumers to an acceptable level.

### Conclusions

This study found that finding an appropriate depression app may be challenging due to the large quantity available. The search results yielded non–depression-specific apps to depression apps at a ratio of 3:1. Over one-quarter of the apps excluded from the study failed to even mention depression in their description or title and exemplify the role of metadata in populating the search results. The lack of reporting of organizational affiliation and content source brings the credibility into question. Whether the content is evidence-based is a whole other issue. This lack of information was most common among symptom management apps, followed by therapeutic treatment and psychoeducation apps. Only medical assessment apps, many of which were based on well-established depression questionnaires, adequately described their sources. As the app phenomenon and health consumerism continue to grow, the user’s ability to find a reliable and credible app may become increasingly difficult. While efforts are underway to populate the marketplace with certifications and professional vetting, this study delineates the need for standards in reporting and for a framework to enable people with depression or other conditions to use proxy measures to assess the legitimacy of apps.

## Multimedia Appendix 1

List of included apps (N=243).

## Abbreviations

CBT: cognitive behavioral therapy
EPDS: Edinburgh Postnatal Depression Scale
ICBT: Internet-based cognitive behavioral therapy
ICT: information and communication technologies
IRR: interrater reliability
PHQ-9: Patient Health Questionnaire
SDS: Zung Self-Rating Depression Scale

## References

[1] Kessler RC, Bromet EJ (2013). The epidemiology of depression across cultures. Annu Rev Public Health.

[2] Murray CJ, Vos T, Lozano R, Naghavi M, Flaxman AD, Michaud C, Ezzati M, Shibuya K, Salomon JA, Abdalla S, Aboyans V, Abraham J, Ackerman I, Aggarwal R, Ahn SY, Ali MK, Alvarado M, Anderson Hr, Anderson LM, Andrews Kg, Atkinson C, Baddour LM, Bahalim AN, Barker-Collo S, Barrero LH, Bartels DH, Basáñez MG, Baxter A, Bell ML, Benjamin EJ, Bennett D, Bernabé E, Bhalla K, Bhandari B, Bikbov B, Bin Abdulhak A, Birbeck G, Black JA, Blencowe H, Blore JD, Blyth F, Bolliger I, Bonaventure A, Boufous S, Bourne R, Boussinesq M, Braithwaite T, Brayne C, Bridgett L, Brooker S, Brooks P, Brugha TS, Bryan-Hancock C, Bucello C, Buchbinder R, Buckle G, Budke CM, Burch M, Burney P, Burstein R, Calabria B, Campbell B, Canter CE, Carabin H, Carapetis J, Carmona L, Cella C, Charlson F, Chen H, Cheng AT, Chou D, Chugh SS, Coffeng LE, Colan SD, Colquhoun S, Colson KE, Condon J, Connor MD, Cooper LT, Corriere M, Cortinovis M, de Vaccaro KC, Couser W, Cowie BC, Criqui MH, Cross M, Dabhadkar KC, Dahiya M, Dahodwala N, Damsere-Derry J, Danaei G, Davis A, De Leo D, Degenhardt L, Dellavalle R, Delossantos A, Denenberg J, Derrett S, Des Jarlais DC, Dharmaratne SD, Dherani M, Diaz-Torne C, Dolk H, Dorsey ER, Driscoll T, Duber H, Ebel B, Edmond K, Elbaz A, Ali SE, Erskine H, Erwin PJ, Espindola P, Ewoigbokhan SE, Farzadfar F, Feigin V, Felson DT, Ferrari A, Ferri CP, Fèvre EM, Finucane MM, Flaxman S, Flood L, Foreman K, Forouzanfar MH, Fowkes FG, Fransen M, Freeman MK, Gabbe BJ, Gabriel SE, Gakidou E, Ganatra HA, Garcia B, Gaspari F, Gillum Rf, Gmel G, Gonzalez-Medina D, Gosselin R, Grainger R, Grant B, Groeger J, Guillemin F, Gunnell D, Gupta R, Haagsma J, Hagan H, Halasa YA, Hall W, Haring D, Haro JM, Harrison JE, Havmoeller R, Hay RJ, Higashi H, Hill C, Hoen B, Hoffman H, Hotez PJ, Hoy D, Huang JJ, Ibeanusi SE, Jacobsen KH, James SL, Jarvis D, Jasrasaria R, Jayaraman S, Johns N, Jonas JB, Karthikeyan G, Kassebaum N, Kawakami N, Keren A, Khoo JP, King CH, Knowlton LM, Kobusingye O, Koranteng A, Krishnamurthi R, Laden F, Lalloo R, Laslett Ll, Lathlean T, Leasher JL, Lee YY, Leigh J, Levinson D, Lim SS, Limb E, Lin JK, Lipnick M, Lipshultz SE, Liu W, Loane M, Ohno SL, Lyons R, Mabweijano J, MacIntyre MF, Malekzadeh R, Mallinger L, Manivannan S, Marcenes W, March L, Margolis DJ, Marks GB, Marks R, Matsumori A, Matzopoulos R, Mayosi BM, McAnulty JH, McDermott MM, McGill N, McGrath J, Medina-Mora ME, Meltzer M, Mensah GA, Merriman TR, Meyer AC, Miglioli V, Miller M, Miller TR, Mitchell PB, Mock C, Mocumbi AO, Moffitt TE, Mokdad AA, Monasta L, Montico M, Moradi-Lakeh M, Moran A, Morawska L, Mori R, Murdoch ME, Mwaniki MK, Naidoo K, Nair MN, Naldi L, Narayan KM, Nelson PK, Nelson RG, Nevitt MC, Newton CR, Nolte S, Norman P, Norman R, O'Donnell M, O'Hanlon S, Olives C, Omer SB, Ortblad K, Osborne R, Ozgediz D, Page A, Pahari B, Pandian JD, Rivero AP, Patten SB, Pearce N, Padilla RP, Perez-Ruiz F, Perico N, Pesudovs K, Phillips D, Phillips MR, Pierce K, Pion S, Polanczyk GV, Polinder S, Pope CA, Popova S, Porrini E, Pourmalek F, Prince M, Pullan RL, Ramaiah KD, Ranganathan D, Razavi H, Regan M, Rehm JT, Rein DB, Remuzzi G, Richardson K, Rivara FP, Roberts T, Robinson C, De Leòn FR, Ronfani L, Room R, Rosenfeld LC, Rushton L, Sacco RL, Saha S, Sampson U, Sanchez-Riera L, Sanman E, Schwebel DC, Scott JG, Segui-Gomez M, Shahraz S, Shepard DS, Shin H, Shivakoti R, Singh D, Singh GM, Singh JA, Singleton J, Sleet DA, Sliwa K, Smith E, Smith Jl, Stapelberg NJ, Steer A, Steiner T, Stolk WA, Stovner LJ, Sudfeld C, Syed S, Tamburlini G, Tavakkoli M, Taylor HR, Taylor JA, Taylor WJ, Thomas B, Thomson WM, Thurston GD, Tleyjeh IM, Tonelli M, Towbin JA, Truelsen T, Tsilimbaris MK, Ubeda C, Undurraga EA, van der Werf MJ, van Os J, Vavilala MS, Venketasubramanian N, Wang M, Wang W, Watt K, Weatherall DJ, Weinstock MA, Weintraub R, Weisskopf MG, Weissman MM, White RA, Whiteford H, Wiebe N, Wiersma ST, Wilkinson JD, Williams HC, Williams SR, Witt E, Wolfe F, Woolf AD, Wulf S, Yeh PH, Zaidi AK, Zheng ZJ, Zonies D, Lopez AD, AlMazroa MA, Memish ZA (2012). Disability-adjusted life years (DALYs) for 291 diseases and injuries in 21 regions, 1990-2010: a systematic analysis for the Global Burden of Disease Study 2010. Lancet.

[3] Mathers C, Fat D, Boerma J (2004). The Global Burden of Disease: 2004 Update.

[4] Titov N, Dear BF, Schwencke G, Andrews G, Johnston L, Craske MG, McEvoy P (2011). Transdiagnostic internet treatment for anxiety and depression: a randomised controlled trial. Behav Res Ther.

[5] Clarke G, Yarborough BJ (2013). Evaluating the promise of health IT to enhance/expand the reach of mental health services. Gen Hosp Psychiatry.

[6] Mohr DC, Ho J, Duffecy J, Baron KG, Lehman KA, Jin L, Reifler D (2010). Perceived barriers to psychological treatments and their relationship to depression. J Clin Psychol.

[7] Mohr DC, Burns MN, Schueller SM, Clarke G, Klinkman M (2013). Behavioral intervention technologies: evidence review and recommendations for future research in mental health. Gen Hosp Psychiatry.

[8] Andrews G, Cuijpers P, Craske MG, McEvoy P, Titov N (2010). Computer therapy for the anxiety and depressive disorders is effective, acceptable and practical health care: a meta-analysis. PLoS One.

[9] Richards D, Richardson T (2012). Computer-based psychological treatments for depression: a systematic review and meta-analysis. Clin Psychol Rev.

[10] Davies EB, Morriss R, Glazebrook C (2014). Computer-delivered and web-based interventions to improve depression, anxiety, and psychological well-being of university students: a systematic review and meta-analysis. J Med Internet Res.

[11] Hooker D, Shen N, Ho K, Ho K, Scott R, Jarvis Selinger S, Novak Lauscher H, Cordiero J (2012). Leveraging community for mHealth research and development. Technology Enabled Knowledge Translation for eHealth.

[12] Luxton DD, McCann RA, Bush NE, Mishkind MC, Reger GM (2011). mHealth for mental health: Integrating smartphone technology in behavioral healthcare. Professional Psychology: Research and Practice.

[13] Kazdin AE, Blase SL (2011). Rebooting Psychotherapy Research and Practice to Reduce the Burden of Mental Illness. Perspectives on Psychological Science.

[14] Donker T, Petrie K, Proudfoot J, Clarke J, Birch MR, Christensen H (2013). Smartphones for smarter delivery of mental health programs: a systematic review. J Med Internet Res.

[15] Proudfoot J (2013). The future is in our hands: the role of mobile phones in the prevention and management of mental disorders. Aust N Z J Psychiatry.

[16] Price M, Yuen EK, Goetter EM, Herbert JD, Forman EM, Acierno R, Ruggiero KJ (2014). mHealth: a mechanism to deliver more accessible, more effective mental health care. Clin Psychol Psychother.

[17] Harrison AM, Goozee R (2014). Psych-related iPhone apps. J Ment Health.

[18] Hundert AS, Huguet A, McGrath PJ, Stinson JN, Wheaton M (2014). Commercially available mobile phone headache diary apps: a systematic review. JMIR Mhealth Uhealth.

[19] Sama PR, Eapen ZJ, Weinfurt KP, Shah BR, Schulman KA (2014). An evaluation of mobile health application tools. JMIR Mhealth Uhealth.

[20] Abroms LC, Padmanabhan N, Thaweethai L, Phillips T (2011). iPhone apps for smoking cessation: a content analysis. Am J Prev Med.

[21] Abroms LC, Lee Westmaas J, Bontemps-Jones J, Ramani R, Mellerson J (2013). A content analysis of popular smartphone apps for smoking cessation. Am J Prev Med.

[22] Choi J, Noh GY, Park DJ (2014). Smoking cessation apps for smartphones: content analysis with the self-determination theory. J Med Internet Res.

[23] Savic M, Best D, Rodda S, Lubman DI (2013). Exploring the focus and experiences of smartphone applications for addiction recovery. J Addict Dis.

[24] Martínez-Pérez B, de la Torre-Díez I, López-Coronado M (2013). Mobile health applications for the most prevalent conditions by the World Health Organization: review and analysis. J Med Internet Res.

[25] Burns MN, Begale M, Duffecy J, Gergle D, Karr CJ, Giangrande E, Mohr DC (2011). Harnessing context sensing to develop a mobile intervention for depression. J Med Internet Res.

[26] Watts S, Mackenzie A, Thomas C, Griskaitis A, Mewton L, Williams A, Andrews G (2013). CBT for depression: a pilot RCT comparing mobile phone vs. computer. BMC Psychiatry.

[27] Kumar S, Nilsen WJ, Abernethy A, Atienza A, Patrick K, Pavel M, Riley WT, Shar A, Spring B, Spruijt-Metz D, Hedeker D, Honavar V, Kravitz R, Lefebvre RC, Mohr DC, Murphy SA, Quinn C, Shusterman V, Swendeman D (2013). Mobile health technology evaluation: the mHealth evidence workshop. Am J Prev Med.

[28] Sharpe R (2012). New England Center for Investigative Reporting.

[29] Steinhubl SR, Muse ED, Topol EJ (2013). Can mobile health technologies transform health care?. JAMA.

[30] Bender JL, Yue RY, To MJ, Deacken L, Jadad AR (2013). A lot of action, but not in the right direction: systematic review and content analysis of smartphone applications for the prevention, detection, and management of cancer. J Med Internet Res.

[31] Landis JR, Koch GG (1977). The measurement of observer agreement for categorical data. Biometrics.

[32] Kroenke K, Spitzer RL, Williams JB (2001). The PHQ-9: validity of a brief depression severity measure. J Gen Intern Med.

[33] Beck AT, Steer RA, Ball R, Ranieri W (1996). Comparison of Beck Depression Inventories -IA and -II in psychiatric outpatients. J Pers Assess.

[34] Yesavage JA, Brink TL, Rose TL, Lum O, Huang V, Adey M, Leirer VO (1982). Development and validation of a geriatric depression screening scale: a preliminary report. J Psychiatr Res.

[35] Gaynes BN, DeVeaugh-Geiss J, Weir S, Gu H, MacPherson C, Schulberg HC, Culpepper L, Rubinow DR (2010). Feasibility and diagnostic validity of the M-3 checklist: a brief, self-rated screen for depressive, bipolar, anxiety, and post-traumatic stress disorders in primary care. Ann Fam Med.

[36] Hollon SD, Kendall PC (1980). Cognitive self-statements in depression: Development of an automatic thoughts questionnaire. Cogn Ther Res.

[37] Radloff LS (1977). The CES-D Scale: A Self-Report Depression Scale for Research in the General Population. Applied Psychological Measurement.

[38] Cox JL, Holden JM, Sagovsky R (1987). Detection of postnatal depression. Development of the 10-item Edinburgh Postnatal Depression Scale. Br J Psychiatry.

[39] Goldberg D, Bridges K, Duncan-Jones P, Grayson D (1988). Detecting anxiety and depression in general medical settings. BMJ.

[40] Rush AJ, Trivedi MH, Ibrahim HM, Carmody TJ, Arnow B, Klein DN, Markowitz JC, Ninan PT, Kornstein S, Manber R, Thase ME, Kocsis JH, Keller MB (2003). The 16-Item Quick Inventory of Depressive Symptomatology (QIDS), clinician rating (QIDS-C), and self-report (QIDS-SR): a psychometric evaluation in patients with chronic major depression. Biol Psychiatry.

[41] Zung W (1965). A Self-Rating Depression Scale. Arch Gen Psychiatry.

[42] Warren C (2013). Google Play hits 1 million apps.

[43] (2014). App store sales top $10 billion in 2013 press release.

[44] van Velsen L, Beaujean DJ, van Gemert-Pijnen JE (2013). Why mobile health app overload drives us crazy, and how to restore the sanity. BMC Med Inform Decis Mak.

[45] Müller RM, Kijl B, Martens JKJ (2011). A comparison of inter-organizational business models of mobile app stores: There is more than open vs. closed. J Theor Appl Electron Commer Res.

[46] Boudreau KJ (2012). Let a Thousand Flowers Bloom? An Early Look at Large Numbers of Software App Developers and Patterns of Innovation. Organization Science.

[47] Sucala M, Schnur JB, Glazier K, Miller SJ, Green JP, Montgomery GH (2013). Hypnosis--there's an app for that: a systematic review of hypnosis apps. Int J Clin Exp Hypn.

[48] West JH, Hall PC, Hanson CL, Barnes MD, Giraud-Carrier C, Barrett J (2012). There's an app for that: content analysis of paid health and fitness apps. J Med Internet Res.

[49] Cowan LT, Van Wagenen SA, Brown BA, Hedin RJ, Seino-Stephan Y, Hall PC, West JH (2013). Apps of steel: are exercise apps providing consumers with realistic expectations?: a content analysis of exercise apps for presence of behavior change theory. Health Educ Behav.

[50] Breton ER, Fuemmeler BF, Abroms LC (2011). Weight loss-there is an app for that! But does it adhere to evidence-informed practices?. Transl Behav Med.

[51] Chomutare T, Fernandez-Luque L, Arsand E, Hartvigsen G (2011). Features of mobile diabetes applications: review of the literature and analysis of current applications compared against evidence-based guidelines. J Med Internet Res.

[52] Benigeri M (2003). Shortcomings of health information on the Internet. Health Promotion International.

[53] Bessière K, Pressman S, Kiesler S, Kraut R (2010). Effects of internet use on health and depression: a longitudinal study. J Med Internet Res.

[54] Reavley NJ, Jorm AF (2011). The quality of mental disorder information websites: a review. Patient Educ Couns.

[55] Deshpande A, Jadad AR (2009). Trying to measure the quality of health information on the internet: is it time to move on?. J Rheumatol.

[56] Crocco AG, Villasis-Keever M, Jadad AR (2002). Analysis of cases of harm associated with use of health information on the internet. JAMA.

[57] Rosser BA, Eccleston C (2011). Smartphone applications for pain management. J Telemed Telecare.

[58] Weaver ER, Horyniak DR, Jenkinson R, Dietze P, Lim MS (2013). "Let's get Wasted!" and Other Apps: Characteristics, Acceptability, and Use of Alcohol-Related Smartphone Applications. JMIR Mhealth Uhealth.

[59] Goyal S, Cafazzo JA (2013). Mobile phone health apps for diabetes management: current evidence and future developments. QJM.

[60] (2011). “Acne cure” mobile app marketers will drop baseless claims under FTC settlements press release.

[61] Ferrero NA, Morrell DS, Burkhart CN (2013). Skin scan: a demonstration of the need for FDA regulation of medical apps on iPhone. J Am Acad Dermatol.

[62] Thompson BM, Brodsky I (2013). Should the FDA regulate mobile medical apps?. BMJ.

[63] (2013). FDA issues final guidance on mobile medical apps press release.

[64] McCarthy M (2013). FDA will not regulate most mobile medical apps. BMJ.

[65] Lippman H (2013). How apps are changing family medicine. J Fam Pract.

[66] Dolan B (2013). Happtique suspends mobile health app certification program.

[67] Boulos MN, Brewer AC, Karimkhani C, Buller DB, Dellavalle RP (2014). Mobile medical and health apps: state of the art, concerns, regulatory control and certification. Online J Public Health Inform.

[68] Deshpande A, Jadad AR (2009). Trying to measure the quality of health information on the internet: is it time to move on?. J Rheumatol.

[69] Nettleton S, Burrows R, O'Malley L (2005). The mundane realities of the everyday lay use of the internet for health, and their consequences for media convergence. Sociol Health Illn.

[70] Dennison L, Morrison L, Conway G, Yardley L (2013). Opportunities and challenges for smartphone applications in supporting health behavior change: qualitative study. J Med Internet Res.

[71] Jung E, Baek C, Lee J (2012). Product survival analysis for the App Store. Mark Lett.

[72] Ryan A, Wilson S (2008). Internet healthcare: do self-diagnosis sites do more harm than good?. Expert Opin Drug Saf.

[73] Christensen H, Reynolds J, Griffiths KM (2011). The use of e-health applications for anxiety and depression in young people: challenges and solutions. Early Interv Psychiatry.

[74] Wade AG (2010). Use of the internet to assist in the treatment of depression and anxiety: a systematic review. Prim Care Companion J Clin Psychiatry.

[75] Henkel V, Mergl R, Kohnen R, Allgaier AK, Möller Hj, Hegerl U (2004). Use of brief depression screening tools in primary care: consideration of heterogeneity in performance in different patient groups. Gen Hosp Psychiatry.

[76] Thombs BD, Coyne JC, Cuijpers P, de Jonge P, Gilbody S, Ioannidis JP, Johnson BT, Patten SB, Turner EH, Ziegelstein RC (2012). Rethinking recommendations for screening for depression in primary care. CMAJ.

[77] Dobbin A, Maxwell M, Elton R (2009). A benchmarked feasibility study of a self-hypnosis treatment for depression in primary care. Int J Clin Exp Hypn.

[78] Montgomery GH, Schnur JB, Kravits K (2013). Hypnosis for cancer care: over 200 years young. CA Cancer J Clin.

[79] Koeser L, Dobbin A, Ross S, McCrone P (2013). Economic evaluation of audio based resilience training for depression in primary care. J Affect Disord.

[80] Frischholz EJ (2013). Antidepressant medications, placebo, and the use of hypnosis in the treatment of depression. Am J Clin Hypn.

[81] Wahbeh H, Calabrese C, Zwickey H (2007). Binaural beat technology in humans: a pilot study to assess psychologic and physiologic effects. J Altern Complement Med.

[82] Huang TL, Charyton C (2008). A comprehensive review of the psychological effects of brainwave entrainment. Altern Ther Health Med.

[83] McCann BS, Landes SJ (2010). Hypnosis in the treatment of depression: considerations in research design and methods. Int J Clin Exp Hypn.

[84] Shih M, Yang YH, Koo M (2009). A meta-analysis of hypnosis in the treatment of depressive symptoms: a brief communication. Int J Clin Exp Hypn.

[85] Høifødt Rs, Lillevoll KR, Griffiths KM, Wilsgaard T, Eisemann M, Waterloo K, Kolstrup N (2013). The clinical effectiveness of web-based cognitive behavioral therapy with face-to-face therapist support for depressed primary care patients: randomized controlled trial. J Med Internet Res.

[86] Johansson R, Andersson G (2012). Internet-based psychological treatments for depression. Expert Rev Neurother.

[87] Lindner P, Ivanova E, Ly KH, Andersson G, Carlbring P (2013). Guided and unguided CBT for social anxiety disorder and/or panic disorder via the Internet and a smartphone application: study protocol for a randomised controlled trial. Trials.

[88] Hedman E, Ljótsson B, Lindefors N (2012). Cognitive behavior therapy via the Internet: a systematic review of applications, clinical efficacy and cost-effectiveness. Expert Rev Pharmacoecon Outcomes Res.

[89] Cramer H, Lauche R, Langhorst J, Dobos G (2013). Yoga for depression: a systematic review and meta-analysis. Depress Anxiety.

[90] Buie E, Blythe M (2013). Spirituality: there’s an app for that! (but not a lot of research). CHI '13 Extended Abstracts on Human Factors in Computing Systems.

[91] Business Wire (2013). Amazon Media Room.

[92] Albrecht UV (2013). Transparency of health-apps for trust and decision making. J Med Internet Res.

[93] Lewis TL (2013). A systematic self-certification model for mobile medical apps. J Med Internet Res.

