# Supplementary material for: Finding a Depression App: A Review and Content Analysis of the Depression App Marketplace
Source: JMIR Mhealth Uhealth. 2015 Feb 16;3(1):e16. doi: 10.2196/mhealth.3713 (PMC4376135; doi:10.2196/mhealth.3713)
Supplement: Supplementary file 1 [file mhealth_v3i1e16_app1.pdf]

| MARKET | APP NAME | LINK |
|---|---|---|
| #APPLE | Am I Depressed | https://itunes.apple.com/ca/app/am-i-depressed/id496508649?mt=8 |
| #APPLE | Anxiety Relief Hypnosis | https://itunes.apple.com/ca/app/anxiety-relief-hypnosis/id593387852?mt=8 |
| #APPLE | Be Happy - Anxiety and Depression | https://itunes.apple.com/ca/app/be-happy-anxiety-depression/id337400410?mt=8 |
| #APPLE | Be Stress Free Fast Stress Test | https://itunes.apple.com/ca/app/be-stress-free-fast-stress/id335319016?mt=8 |
| #APPLE | Beat Depression | https://itunes.apple.com/ca/app/beat-depression/id602796385?mt=8 |
| #APPLE | Better Mood Tracker | https://itunes.apple.com/ca/app/better-mood-tracker/id572360495?mt=8 |
| #APPLE | Brain Freqz - Depression Relief | https://itunes.apple.com/ca/app/brainfreqz-depression-relief/id306244990?mt=8 |
| #APPLE | Deal with Depression | https://itunes.apple.com/ca/app/deal-with-depression/id513562379?mt=8 |
| #APPLE | Dealing With Negative Emotions | https://itunes.apple.com/ca/app/dealing-negative-emotions/id464359078?mt=8 |
| #APPLE | Defeat Depression | https://itunes.apple.com/ca/app/defeat-depression/id399584993?mt=8 |
| #APPLE | Depressed | https://itunes.apple.com/ca/app/depressed/id545930572?mt=8 |
| #APPLE | Depression - Plain English | https://itunes.apple.com/ca/app/depression-plain-english-health/id402704764?mt=8 |
| #APPLE | Depression By ASKONE | https://itunes.apple.com/ca/app/idepression/id377702166?mt=8 |
| #APPLE | Depression Calculator | https://itunes.apple.com/ca/app/depression-calculator/id517937129?mt=8 |
| #APPLE | Depression Check | https://itunes.apple.com/ca/app/depressioncheck/id398170644?mt=8 |
| #APPLE | Depression Consultant | https://itunes.apple.com/ca/app/depression-consultant/id502969441?mt=8 |
| #APPLE | Depression Cure - The Free 12 week course | https://itunes.apple.com/us/app/depression-cure-free-12-week/id546948783?mt=8 |
| #APPLE | Depression Fighter | https://itunes.apple.com/ca/app/depression-fighter-practical/id471879726?mt=8 |
| #APPLE | Depression Guide | https://itunes.apple.com/ca/app/depression-guide/id377887296?mt=8 |
| #APPLE | Depression Help Brainwave | https://itunes.apple.com/ca/app/depression-help-brainwave/id416501896?mt=8 |
| #APPLE | Depression Jorunal | https://itunes.apple.com/ca/app/depression- |

| MARKET | APP NAME | LINK |
| --- | --- | --- |
| | | journal/id320585382?mt=8 |
| #APPLE | Depression Monitor | https://itunes.apple.com/ca/app/depression-monitor/id528600269?mt=8 |
| #APPLE | Depression Mood Monitor | https://itunes.apple.com/ca/app/depression-mood-monitor/id467815397?mt=8 |
| #APPLE | Depression Relief and Mood - HappyApp | https://itunes.apple.com/ca/app/depression-relief-mood-support/id464669769?mt=8 |
| #APPLE | Depression Screeing | http://bit.ly/10bie4i |
| #APPLE | Depression Screening Pro | https://itunes.apple.com/ca/app/depression-screening-pro/id587464255?mt=8 |
| #APPLE | Depression Test & Tracker | https://itunes.apple.com/ca/app/depression-test-tracker/id341509748?mt=8 |
| #APPLE | Depression Treatment | https://itunes.apple.com/ca/app/depression-treatment/id345598701?mt=8 |
| #APPLE | DepressPill Game for Happy | https://itunes.apple.com/ca/app/depresspill-game-for-happy/id420375805?mt=8 |
| #APPLE | DepressPill LITE - Game for Happy Habit | https://itunes.apple.com/ca/app/depresspill-lite-game-for/id424161567?mt=8 |
| #APPLE | DigitalMeds | https://itunes.apple.com/ca/app/digitalmeds/id571275152?mt=8 |
| #APPLE | DigitalMeds Lite | https://itunes.apple.com/ca/app/digitalmeds-lite/id571303484?mt=8 |
| #APPLE | Do I Have Depression | https://itunes.apple.com/ca/app/do-i-have-depression/id460556669?mt=8 |
| #APPLE | Do I Have Depression (HD) | https://itunes.apple.com/ca/app/do-i-have-depression-hd/id463800297?mt=8 |
| #APPLE | Duas for Depression and Agony | https://itunes.apple.com/ca/app/duas-for-depression-and-agony/id384080076?mt=8 |
| #APPLE | Easing Anxiety and Depression | https://itunes.apple.com/ca/app/easing-anxiety-depression/id529562246?mt=8 |
| #APPLE | eCBT Mood | https://itunes.apple.com/ca/app/ecbt-mood/id324060472?mt=8 |
| #APPLE | eLifeguard App | https://itunes.apple.com/ca/app/elifeguard-app/id499947494?mt=8 |
| #APPLE | Fix Depression | https://itunes.apple.com/ca/app/fix-depression/id489451675?mt=8 |
| #APPLE | Fix Depression (Lite) | https://itunes.apple.com/ca/app/fix-depression-lite/id505786709?mt=8 |
| #APPLE | Geriatric Depression Scale | https://itunes.apple.com/ca/app/geriatric-depression-scale/id433446297?mt=8 |
| #APPLE | Habit Changer Feeling Happier | https://itunes.apple.com/ca/app/habit-changer-feeling- |

| MARKET | APP NAME | LINK |
|---|---|---|
| | | happier/id376949733?mt=8 |
| #APPLE | Happy App - Beat Depression | https://itunes.apple.com/ca/app/happy-app-beat-depression/id449889863?mt=8 |
| #APPLE | How to understand and Have a Happy Mind | https://itunes.apple.com/ca/app/how-to-understand-have-happy/id339463419?mt=8 |
| #APPLE | I Smile Today | https://itunes.apple.com/ca/app/i-smile-today/id376739061?mt=8 |
| #APPLE | iCouch CBT | https://itunes.apple.com/ca/app/icouch-cbt/id446115508?mt=8 |
| #APPLE | iCounselor Depression | https://itunes.apple.com/ca/app/icounselor-depression/id346703658?mt=8 |
| #APPLE | iDepression Tracker | https://itunes.apple.com/ca/app/idepressiontracker/id503787796?mt=8 |
| #APPLE | iMind & Mood | https://itunes.apple.com/ca/app/imind-mood/id515748296?mt=8 |
| #APPLE | iSelfhelp - Depression | https://itunes.apple.com/ca/app/iselfhelp-depression/id481240621?mt=8 |
| #APPLE | iSelfhelp - Mental Health Test | https://itunes.apple.com/ca/app/iselfhelp-mental-health-test/id483328770?mt=8 |
| #APPLE | iSelfhep Stockbroker | https://itunes.apple.com/ca/app/iselfhelp-stockbrokers-psychtest/id491679837?mt=8 |
| #APPLE | Jesus Children Business Card | https://itunes.apple.com/ca/app/jesus-children-business-card/id433806190?mt=8 |
| #APPLE | Life Improvement Tips | https://itunes.apple.com/ca/app/life-improvement-tips/id385465065?mt=8 |
| #APPLE | Living out of Darkness | https://itunes.apple.com/ca/app/living-out-of-darkness/id336931147?mt=8 |
| #APPLE | Mental Elf | https://itunes.apple.com/ca/app/mental-elf/id542192849?mt=8 |
| #APPLE | Mental Rec | https://itunes.apple.com/ca/app/mental-rec/id391071946?mt=8 |
| #APPLE | Mentally Fit | https://itunes.apple.com/ca/app/mentally-fit/id548304170?mt=8 |
| #APPLE | Mood Sentry | https://itunes.apple.com/ca/app/moodsentry/id576269929?mt=8 |
| #APPLE | Mood Watch | https://itunes.apple.com/ca/app/mood-watch/id590103778?mt=8 |
| #APPLE | MoodMaster Anti-Depression | https://itunes.apple.com/ca/app/moodmaster-anti-depression/id463445969?mt=8 |
| #APPLE | MusicClinic: 4 In 1 | https://itunes.apple.com/ca/app/musicclinic-4-in-1/id336264547?mt=8 |

| MARKET | APP NAME | LINK |
|---|---|---|
| #APPLE | MusicClinic: DepressionManagement | https://itunes.apple.com/ca/app/musicclinic-depressionmanagement/id329223356?mt=8 |
| #APPLE | MusicClinic: Sleep | https://itunes.apple.com/ca/app/music-clinic-sleep/id390019591?mt=8 |
| #APPLE | my Smiley Calendar | https://itunes.apple.com/ca/app/my-smiley-calendar/id575454311?mt=8 |
| #APPLE | MyMentalQuiz | https://itunes.apple.com/ca/app/mymentalquiz/id425153883?mt=8 |
| #APPLE | MyMentalState for iPhone | https://itunes.apple.com/ca/app/mymentalstate-for-iphone/id431194260?mt=8 |
| #APPLE | Operation Reach Out | https://itunes.apple.com/ca/app/operation-reach-out/id478899653?mt=8 |
| #APPLE | Optimism | https://itunes.apple.com/ca/app/optimism/id352262677?mt=8 |
| #APPLE | Overcoming Depression | https://itunes.apple.com/ca/app/overcoming-depression/id402402292?mt=8 |
| #APPLE | Pain & Depression Relief | https://itunes.apple.com/ca/app/pain-depression-relief-ambiscience/id441043960?mt=8 |
| #APPLE | PHQ-9 | https://itunes.apple.com/ca/app/phq-9/id533001786?mt=8 |
| #APPLE | PocketShrink Depression 1.0 | https://itunes.apple.com/ca/app/pocketshrink-depression-1.0/id564119019?mt=8 |
| #APPLE | Postnatal Depression Test | https://itunes.apple.com/ca/app/postnatal-depression-test/id598368749?mt=8 |
| #APPLE | Postnatal Depression Test PRO | https://itunes.apple.com/ca/app/postnatal-depression-test/id600038327?mt=8 |
| #APPLE | PPD Gone! | https://itunes.apple.com/ca/app/ppd-gone!/id529141505?mt=8 |
| #APPLE | Psychiatry (Understanding Disease) | https://itunes.apple.com/ca/app/psychiatry-understanding-disease/id411303269?mt=8 |
| #APPLE | Sad Scale | https://itunes.apple.com/ca/app/sad-scale/id308056730?mt=8 |
| #APPLE | Sad Scale Lite | https://itunes.apple.com/ca/app/sad-scale-lite/id332139818?mt=8 |
| #APPLE | Self Esteem Hypnosis | https://itunes.apple.com/ca/app/self-esteem-hypnosis/id501209110?mt=8 |
| #APPLE | Senior Mood Assessment | https://itunes.apple.com/ca/app/senior-mood-assessment-depressed/id430169679?mt=8 |
| #APPLE | Seven Minute Stress Cure | https://itunes.apple.com/ca/app/seven-minute-stress-cure/id342816465?mt=8 |
| #APPLE | SleepNotes - Lifestyle | https://itunes.apple.com/ca/app/sleepnotes-lifestyle- |

| MARKET | APP NAME | LINK |
|---|---|---|
| | | administrator/id574187245?mt=8 |
| #APPLE | STAT Depression Screening | http://bit.ly/YUzsy5 |
| #APPLE | Stop Depression and Anxiety | https://itunes.apple.com/ca/app/stop-depression-anxiety-relax/id464293557?mt=8 |
| #APPLE | Tap MotivateMe | https://itunes.apple.com/ca/app/tap-motivateme/id543876271?mt=8 |
| #APPLE | The Mindful Way Thought | https://itunes.apple.com/ca/app/mindful-way-through-depression/id526530776?mt=8 |
| #APPLE | VirtualClinic - The Get happy | https://itunes.apple.com/ca/app/virtualclinic-get-happy-program/id493461510?mt=8 |
| #APPLE | Your Life with Depression | https://itunes.apple.com/ca/app/your-life-depression-patient/id561909060?mt=8 |
| #APPLE | Zung | https://itunes.apple.com/ca/app/zung/id440333901?mt=8 |
| #BB | Defeat Depression | http://appworld.blackberry.com/webstore/content/22053878/?countrycode=CA |
| #BB | Depression Test PHQ-9 | http://appworld.blackberry.com/webstore/content/20399226/?countrycode=CA |
| #BB | Help Yourself: Depression | http://appworld.blackberry.com/webstore/content/32701/?countrycode=CA |
| #BB | National Institute of Health Depression Consultant | http://appworld.blackberry.com/webstore/content/1610/?countrycode=CA |
| #BB | Teach Yourself Cognitive Behavioural Therapy | http://appworld.blackberry.com/webstore/content/22017176/?countrycode=CA |
| #BB | The Depression Predictor | http://appworld.blackberry.com/webstore/content/23377875/?countrycode=CA |
| #GOOG | Am I Depressed? | https://play.google.com/store/apps/details?id=com.softomatrix.amidepressed&feature=search_result#?t=W251bGwsMSwxLDEsImNvbS5zb2Z0b21hdHJpeC5hbWlkZXByZXNzZWQiXQ |
| #GOOG | Anti-Depression and Anxiety | https://play.google.com/store/apps/details?id=com.wAntiDepressionAudioRhythms&feature=search_result#?t=W251bGwsMSwxLDEsImNvbS53QW50aURlcHJlc3Npb25BdWRpb1JoeXRobXMiXQ |
| #GOOG | Anti-Depression Grocery List | https://play.google.com/store/apps/details?id=air.com.lisieremedia.AntiDepressionGroceryList&feature=search_result#?t=W251bGwsMSwxLDEsImFpci5jb20ubGlzaWVyZW1lZGlhLkFudGlEZXByZXNzaW9uR3JvY2VyeUxpc3QiXQ |

| MARKET | APP NAME | LINK |
|---|---|---|
| #GOOG | Anxiety & Panic Relief Therapy | https://play.google.com/store/apps/details?id=mobi.brapp.ashicra&feature=search_result#?t=W251bGwsMSwxLDEsIm1vYmkuYnJhcHAuYXNoaWNyYSJd |
| #GOOG | Anxiety and Depression | https://play.google.com/store/apps/details?id=com.guide.Anxiety.and.Depression&feature=search_result#?t=W251bGwsMSwxLDEsImNvbS5ndWlkZS5BbnhpZXR5LmFuZC5EZXByZXNzaW9uIl0. |
| #GOOG | Anxiety and Depression | https://play.google.com/store/apps/details?id=appinventor.ai_appcreator1970.Anxiety_and_Depression&feature=search_result#?t=W251bGwsMSwxLDEsImFwcGludmVudG9yLmFpX2FwcGNyZWF0b3IxOTcwLkFueGlldHlfYW5kX0RlcHJlc3Npb24iXQ |
| #GOOG | Anxiety and Depression | https://play.google.com/store/apps/details?id=de.actsmartware.app000005986&feature=search_result#?t=W251bGwsMSwxLDEsImRlLmFjdHNtYXJ0d2FyZS5hcHAwMDAwMDU5ODYiXQ |
| #GOOG | Anxiety and Depression 101 | https://play.google.com/store/apps/details?id=com.myappbuilder.AnxietyAndDepression101&feature=search_result#?t=W251bGwsMSwxLDEsImNvbS5teWFwcGJ1aWxkZXIuQW54aWV0eUFuZERlcHJlc3Npb24xMDEiXQ.. |
| #GOOG | Anxiety Depression | https://play.google.com/store/apps/details?id=com.Depression21.book.AOTFJFHBTTOABNBJKO&feature=search_result#?t=W251bGwsMSwxLDEsImNvbS5EZXByZXNzaW9uMjEuYm9vay5BT1RGSkZIQlRUT0FCTkJKS08iXQ.. |
| #GOOG | Are you at risk for depression? | https://play.google.com/store/apps/details?id=com.sounddepressiontreatment.subliminal&feature=search_result#?t=W251bGwsMSwxLDEsImNvbS5zb3VuZGRlcHJlc3Npb250cmVhdG1lbnQuc3VibGltaW5hbCJd |
| #GOOG | Audio Book Anxiety and Depression | https://play.google.com/store/apps/details?id=anace.com.audiobooks.anxiety_depression&feature=search_result#?t=W251bGwsMSwxLDEsImFuYWNlLmNvbS5hdWRpb2Jvb2tzLmFueGlldHlfZGVwcmVzc2lvbiJd |
| #GOOG | Automatic Positive Thought Generator | https://play.google.com/store/apps/details?id=appinventor.ai_jonfinlay.APT&feature=search_result |

| MARKET | APP NAME | LINK |
|---|---|---|
| | | #?t=W251bGwsMSwxLDEsImFwcGludmVudG9yLmFpX2pvbmZpbmxheS5BUFQiXQ |
| #GOOG | Beat Depression | https://play.google.com/store/apps/details?id=beat.depression&feature=search_result#?t=W251bGwsMSwxLDEsImJlYXQuZGVwcmVzc2lvbiJd |
| #GOOG | Beat Depression (Smart Guide) | https://play.google.com/store/apps/details?id=com.pixidapp.beatdepressionzip&feature=search_result#?t=W251bGwsMSwxLDEsImNvbS5waXhpZGFwcC5iZWF0ZGVwcmVzc2lvbnppcCJd |
| #GOOG | Beat Depression Hypnosis System | https://play.google.com/store/apps/details?id=com.andromo.dev9483.app146203&feature=search_result#?t=W251bGwsMSwxLDEsImNvbS5hbmRyb21vLmRldjk0ODMuYXBwMTQ2MjAzIl0 |
| #GOOG | Beat Depression with Self-Help | https://play.google.com/store/apps/details?id=com.digimediaapps.Beat_Depression_with_Self_Help_Techniques&feature=search_result#?t=W251bGwsMSwxLDEsImNvbS5kaWdpbWVkaWFhcHBzLkJlYXRfRGVwcmVzc2lvbl93aXRoX1NlbGZfSGVscF9UZWNobmlxdWVzIl0 |
| #GOOG | Beautiful Rain Relaxation | https://play.google.com/store/apps/details?id=com.mozinc.beautifulrain&feature=search_result#?t=W251bGwsMSwxLDEsImNvbS5tb3ppbmMuYmVhdXRpZnVscmFpbiJd |
| #GOOG | Beck Depression Inventory | https://play.google.com/store/apps/details?id=Dardiries.BDI.BDI&feature=search_result#?t=W251bGwsMSwxLDEsIkRhcmRpcmllcy5CREkuQkRJIl0 |
| #GOOG | Beck Depression Inventory BD12 | https://play.google.com/store/apps/details?id=Dardiries.BDI2.BDI2&feature=search_result#?t=W251bGwsMSwxLDEsIkRhcmRpcmllcy5CREkyLkJESTIiXQ.. |
| #GOOG | Bi-Polar Disorder | https://play.google.com/store/apps/details?id=de.actsmartware.app000006964&feature=search_result#?t=W251bGwsMSwxLDEsImRlLmFjdHNtYXJ0d2FyZS5hcHAwMDAwMDY5NjQiXQ |
| #GOOG | Bipolar Disorder! | https://play.google.com/store/apps/details?id=com.kinetixapps.bipolardisorder&feature=search_result#? |

| MARKET | APP NAME | LINK |
|---|---|---|
| | | t=W251bGwsMSwxLDEsImNvbS5raW5ldGl4YXBwcy5iaXBvbGFyZGlzb3JkZXIiXQ |
| #GOOG | Blue Light | https://play.google.com/store/apps/details?id=droid.bluelight&feature=search_result#?t=W251bGwsMSwxLDEsImRyb2lkLmJsdWVsaWdodCJd |
| #GOOG | Blue Wallpapers | https://play.google.com/store/apps/details?id=com.blue.wallpapers&feature=search_result#?t=W251bGwsMSwxLDEsImNvbS5ibHVlLndhbGxwYXBlcnMiXQ |
| #GOOG | CBT Referee | https://play.google.com/store/apps/details?id=com.cbtreferee&feature=search_result#?t=W251bGwsMSwxLDEsImNvbS5jYnRyZWZlcmVlIl0 |
| #GOOG | Chantu Bantu Jokes | https://play.google.com/store/apps/details?id=com.mandeeprekhi.santabanta&feature=search_result#?t=W251bGwsMSwxLDEsImNvbS5tYW5kZWVwcmVraGkuc2FudGFiYW50YSJd |
| #GOOG | Cognitive Syles CBT Test | https://play.google.com/store/apps/details?id=com.excelatlife.cbttest&feature=search_result#?t=W251bGwsMSwxLDEsImNvbS5leGNlbGF0bGlmZS5jYnR0ZXN0Il0. |
| #GOOG | Comic Mask | https://play.google.com/store/apps/details?id=com.olaworks.automask.comic&feature=search_result#?t=W251bGwsMSwxLDEsImNvbS5vbGF3b3Jrcy5hdXRvbWFzay5jb21pYyJd |
| #GOOG | Comic Mask Lite | https://play.google.com/store/apps/details?id=com.olaworks.automask.comic_lite&feature=search_result#?t=W251bGwsMSwxLDEsImNvbS5vbGF3b3Jrcy5hdXRvbWFzay5jb21pY19saXRlIl0 |
| #GOOG | Crack Screen | https://play.google.com/store/apps/details?id=com.hzwp.BrokenScreen&feature=search_result#?t=W251bGwsMSwxLDEsImNvbS5oendwLkJyb2tlblNjcmVlbiJd |
| #GOOG | Dealing with Depression | https://play.google.com/store/apps/details?id=com.wDepression&feature=search_result#?t=W251bGwsMSwxLDEsImNvbS53RGVwcmVzc2lvbiJd |
| #GOOG | Dealing with Depression | https://play.google.com/store/apps/details?id=com.wDealingwithDepression&feature=search_result |

| MARKET | APP NAME | LINK |
|---|---|---|
| | | #?<br>t=W251bGwsMSwxLDEsImNvbS53RGVhbGluZ3dpdGhEZXByZXNzaW9uIl0 |
| #GOOG | Defeat Depression | https://play.google.com/store/apps/details?id=com.appmk.defeatdepression.AOTMEDHCLGYSDFYXC&feature=search_result#?t=W251bGwsMSwxLDEsImNvbS5hcHBtay5kZWZlYXRkZXByZXNzaW9uLkFPVE1FREhDTEdZU0RGWVhDIl0 |
| #GOOG | Depressed? | https://play.google.com/store/apps/details?id=com.foncannoninc.depression&feature=search_result#?t=W251bGwsMSwxLDEsImNvbS5mb25jYW5ub25pbmMuZGVwcmVzc2lvbiJd |
| #GOOG | Depression | https://play.google.com/store/apps/details?id=air.com.asdspecialist.depression&feature=search_result#?t=W251bGwsMSwxLDEsImFpci5jb20uYXNkc3BlY2lhbGlzdC5kZXByZXNzaW9uIl0 |
| #GOOG | Depression | https://play.google.com/store/apps/details?id=com.simpaddico.flashcards.depression&feature=search_result#?t=W251bGwsMSwxLDEsImNvbS5zaW1wYWRkaWNvLmZsYXNoY2FyZHMuZGVwcmVzc2lvbiJd |
| #GOOG | Depression | https://play.google.com/store/apps/details?id=com.focusmedica.depression&feature=search_result#?t=W251bGwsMSwxLDEsImNvbS5mb2N1c21lZGljYS5kZXByZXNzaW9uIl0 |
| #GOOG | Depression | https://play.google.com/store/apps/details?id=com.vertexmind.DEPRESSIONAMisunderstoodDISEASE.ebw&feature=search_result#?t=W251bGwsMSwxLDEsImNvbS52ZXJ0ZXhtaW5kLkRFUFJFU1NJT05BTWlzdW5kZXJzdG9vZERJU0VBU0UuZWJ3Il0. |
| #GOOG | Depression 101 by WAGmob | https://play.google.com/store/apps/details?id=com.quizmine.depression&feature=search_result#?t=W251bGwsMSwxLDEsImNvbS5xdWl6bWluZS5kZXByZXNzaW9uIl0. |
| #GOOG | Depression Advice | https://play.google.com/store/apps/details?id=com.appmakr.app356528&feature=search_result#?t=W251bGwsMSwxLDEsImNvbS5hcHBtYWtyLmFwcDM1NjUyOCJd |

| MARKET | APP NAME | LINK |
|---|---|---|
| #GOOG | Depression and Anxiety | https://play.google.com/store/apps/details?id=com.a10187385695068b15b36a349a.a45257883a&feature=search_result#?t=W251bGwsMSwxLDEsImNvbS5hMTAxODczODU2OTUwNjhiMTViMzZhMzQ5YS5hNDUyNTc4ODNhIl0. |
| #GOOG | Depression and Heart Disease | https://play.google.com/store/apps/details?id=com.yogi.yogidepressionandcoronaryheartdisease&feature=search_result#?t=W251bGwsMSwxLDEsImNvbS55b2dpLnlvZ2lkZXByZXNzaW9uYW5kY29yb25hcnloZWFydGRpc2Vhc2UiXQ.. |
| #GOOG | Depression by Giovani Lordi | https://play.google.com/store/apps/details?id=com.imobilize.depressiongl&feature=search_result#?t=W251bGwsMSwxLDEsImNvbS5pbW9iaWxpemUuZGVwcmVzc2lvbmdsIl0 |
| #GOOG | Depression CBT Self-Help Guide | https://play.google.com/store/apps/details?id=com.excelatlife.depression&feature=search_result#?t=W251bGwsMSwxLDEsImNvbS5leGNlbGF0bGlmZS5kZXByZXNzaW9uIl0 |
| #GOOG | Depression Connect | https://play.google.com/store/apps/details?id=com.alliancehealth.depressionconnect&feature=search_result#?t=W251bGwsMSwxLDEsImNvbS5hbGxpYW5jZWhlYWx0aC5kZXByZXNzaW9uY29ubmVjdCJd |
| #GOOG | Depression Diagnosis | https://play.google.com/store/apps/details?id=com.easydiagnosis.depression&feature=search_result#?t=W251bGwsMSwxLDEsImNvbS5lYXN5ZGlhZ25vc2lzLmRlcHJlc3Npb24iXQ.. |
| #GOOG | Depression Dog | https://play.google.com/store/apps/details?id=com.amf.depressiondog&feature=search_result#?t=W251bGwsMSwxLDEsImNvbS5hbWYuZGVwcmVzc2lvbmRvZyJd |
| #GOOG | Depression Health Brainwave Lite | https://play.google.com/store/apps/details?id=imoblife.depressionhelpbrainwave.lite&feature=search_result#?t=W251bGwsMSwxLDEsImltb2JsaWZlLmRlcHJlc3Npb25oZWxwYnJhaW53YXZlLmxpdGUiXQ |
| #GOOG | Depression Help | https://play.google.com/store/apps/details?id=stop.depression.tips&feature=search_result#?t=W251bGwsMSwxLDEsInN0b3AuZGVwcmVzc2lvbi50aXBzIl0. |

| MARKET | APP NAME | LINK |
|---|---|---|
| #GOOG | Depression Help | https://play.google.com/store/apps/details?id=com.a2009682521512910 8f22d668a.a25813790a&feature=search_result#?t=W251bGwsMSwxLDEsImNvbS5hMjAwOTY4MjUyMTUxMjkxMDhmMjJkNjY4YS5hMjU4MTM3OTBhIl0 |
| #GOOG | Depression Help Brainwave | https://play.google.com/store/apps/details?id=imoblife.depressionhelpbrainwave.full&feature=search_result#?t=W251bGwsMSwxLDEsImltb2JsaWZlLmRlcHJlc3Npb25oZWxwYnJhaW53YXZlLmZ1bGwiXQ |
| #GOOG | Depression Home Remedies | https://play.google.com/store/apps/details?id=com.max.maxdepressionhomeremedies&feature=search_result#?t=W251bGwsMSwxLDEsImNvbS5tYXgubWF4ZGVwcmVzc2lvbmhvbWVyZW1lZGllcyJd |
| #GOOG | Depression Inventory | https://play.google.com/store/apps/details?id=com.fnirt.persin_depression&feature=search_result#?t=W251bGwsMSwxLDEsImNvbS5mbmlydC5wZXJzaW5fZGVwcmVzc2lvbiJd |
| #GOOG | Depression Preview | https://play.google.com/store/apps/details?id=com.vertexmind.DEPRESSIONAMisunderstoodDISEASEPreview.ebw&feature=search_result#?t=W251bGwsMSwxLDEsImNvbS52ZXJ0ZXhtaW5kLkRFUFJFU1NJT05BTWlzdW5kZXJzdG9vZERJU0VBU0VQcmV2aWV3LmVidyJd |
| #GOOG | Depression Survey | https://play.google.com/store/apps/details?id=com.webbresearch.ulcersurvey&feature=search_result#?t=W251bGwsMSwxLDEsImNvbS53ZWJicmVzZWFyY2gudWxjZXJzdXJ2ZXkiXQ.. |
| #GOOG | Depression Symptoms | https://play.google.com/store/apps/details?id=com.amgapp.depressionsymptoms&feature=search_result#?t=W251bGwsMSwxLDEsImNvbS5hbWdhcHAuZGVwcmVzc2lvbnN5bXB0b21zIl0 |
| #GOOG | Depression Tame Subliminal Free | https://play.google.com/store/apps/details?id=com.t3apps.tamedepressionfree&feature=search_result#?t=W251bGwsMSwxLDEsImNvbS50M2FwcHMudGFtZWRlcHJlc3Npb25mcmVlIl0. |
| #GOOG | Depression Tamer Subliminal | https://play.google.com/store/apps/details? |

| MARKET | APP NAME | LINK |
|---|---|---|
| | | id=com.t3apps.tamedepression&feature=search_result#?t=W251bGwsMSwxLDEsImNvbS50M2FwcHMudGFtZWRlcHJlc3Npb24iXQ |
| #GOOG | Depression Test | https://play.google.com/store/apps/details?id=nl.japps.android.depressiontest&feature=search_result#?t=W251bGwsMSwxLDEsIm5sLmphcHBzLmFuZHJvaWQuZGVwcmVzc2lvbnRlc3QiXQ.. |
| #GOOG | Depression Test Demystified | https://play.google.com/store/apps/details?id=com.wDepressionTestDemystified&feature=search_result#?t=W251bGwsMSwxLDEsImNvbS53RGVwcmVzc2lvblRlc3REZW15c3RpZmllZCJd |
| #GOOG | Depression Treatment | https://play.google.com/store/apps/details?id=com.wDepressionTreatment&feature=search_result#?t=W251bGwsMSwxLDEsImNvbS53RGVwcmVzc2lvblRyZWF0bWVudCJd |
| #GOOG | Drip of Insight Relaxation | https://play.google.com/store/apps/details?id=com.mozinc.dripofinsight&feature=search_result#?t=W251bGwsMSwxLDEsImNvbS5tb3ppbmMuZHJpcG9maW5zaWdodCJd |
| #GOOG | eMood Bipolar Mood Tracker | https://play.google.com/store/apps/details?id=my.tracker&feature=search_result#?t=W251bGwsMSwxLDEsIm15LnRyYWNrZXIiXQ |
| #GOOG | End Anxiety Brainwave Audio | https://play.google.com/store/apps/details?id=com.wReduceAnxietyNow&feature=search_result#?t=W251bGwsMSwxLDEsImNvbS53UmVkdWNlQW54aWV0eU5vdyJd |
| #GOOG | End Depression Audio Rhythms | https://play.google.com/store/apps/details?id=com.wCombatDepressionAudio&feature=search_result#?t=W251bGwsMSwxLDEsImNvbS53Q29tYmF0RGVwcmVzc2lvbkF1ZGlvIl0 |
| #GOOG | Extreme Motivational Quotes | https://play.google.com/store/apps/details?id=com.via3apps.aextrememem872&feature=search_result#?t=W251bGwsMSwxLDEsImNvbS52aWEzYXBwcy5hZXh0cmVtZW04NzIiXQ |
| #GOOG | Fight Depression: 8 Tips | https://play.google.com/store/apps/details?id=com.kinetixapps.fightdepression&feature=search_res |

| MARKET | APP NAME | LINK |
|---|---|---|
| | | ult#?<br>t=W251bGwsMSwxLDEsImNvbS5raW5ldGl4YXBwcy5maWdodGRlcHJlc3Npb24iXQ.. |
| #GOOG | Ganesh Mantra | https://play.google.com/store/apps/details?<br>id=com.bhaee.ganesh&feature=search_result#?<br>t=W251bGwsMSwxLDEsImNvbS5iaGFlZS5nYW5lc2giXQ |
| #GOOG | Geriatric Depression Scale | https://play.google.com/store/apps/details?<br>id=appinventor.ai_yesavage.GeriatricDepressionScale&feature=search_result#?<br>t=W251bGwsMSwxLDEsImFwcGludmVudG9yLmFpX3llc2F2YWdlLkdlcmlhdHJpY0RlcHJlc3Npb25TY2FsZSJd |
| #GOOG | Happy App | https://play.google.com/store/apps/details?<br>id=com.ib.happyapp.activity&feature=search_result#?<br>t=W251bGwsMSwxLDEsImNvbS5pYi5oYXBweWFwcC5hY3Rpdml0eSJd |
| #GOOG | Hatha Yoga App | https://play.google.com/store/apps/details?<br>id=com.a178724170250ae6a504dfff4a.a58313051a&feature=search_result#?<br>t=W251bGwsMSwxLDEsImNvbS5hMTc4NzI0MTcwMjUwYWU2YTUwNGRmZmY0YS5hNTgzMTMwNTFhIl0. |
| #GOOG | Healing Mind Relaxation | https://play.google.com/store/apps/details?<br>id=com.mozinc.healingmind&feature=search_result#?<br>t=W251bGwsMSwxLDEsImNvbS5tb3ppbmMuaGVhbGluZ21pbmQiXQ.. |
| #GOOG | Health Depression Hypnosis | https://play.google.com/store/apps/details?<br>id=com.hypnotransformations.healdepression&feature=search_result#?<br>t=W251bGwsMSwxLDEsImNvbS5oeXBub3RyYW5zZm9ybWF0aW9ucy5oZWFsZGVwcmVzc2lvbiJd |
| #GOOG | Jesus Blessing You | https://play.google.com/store/apps/details?<br>id=com.sou.christ&feature=search_result#?<br>t=W251bGwsMSwxLDEsImNvbS5zb3UuY2hyaXN0Il0 |
| #GOOG | Jesus Christ Live Wallpaper | https://play.google.com/store/apps/details?<br>id=com.livewallpaper.lordjesus&feature=search_result#?<br>t=W251bGwsMSwxLDEsImNvbS5saXZld2FsbHBhcGVyLmxvcmRqZXN1cyJd |
| #GOOG | Joker | https://play.google.com/store/apps/details?<br>id=com.mezco.Joker&feature=search_result#?<br>t=W251bGwsMSwxLDEsImNvbS5tZXpjby5Kb2tlciJd |

| MARKET | APP NAME | LINK |
|---|---|---|
| #GOOG | Learn About Depression | https://play.google.com/store/apps/details?id=com.ProjectNotAloneDepressionModule&feature=search_result#?t=W251bGwsMSwxLDEsImNvbS5Qcm9qZWN0Tm90QWxvbmVEZXByZXNzaW9uTW9kdWxlIl0 |
| #GOOG | Let Go | https://play.google.com/store/apps/details?id=com.araaya.letgo&feature=search_result#?t=W251bGwsMSwxLDEsImNvbS5hcmFheWEubGV0Z28iXQ |
| #GOOG | Living with BiPolar Disorder | https://play.google.com/store/apps/details?id=com.LivingWithBiPolarDisorder.book.AOTEOFOYEKEARAIXMZ&feature=search_result#?t=W251bGwsMSwxLDEsImNvbS5MaXZpbmdXaXRoQmlQb2xhckRpc29yZGVyLmJvb2suQU9URU9GT1lFS0VBUkFJWE1aIl0 |
| #GOOG | Lucid Mind Relaxation | https://play.google.com/store/apps/details?id=com.mozinc.lucidmind&feature=search_result#?t=W251bGwsMSwxLDEsImNvbS5tb3ppbmMubHVjaWRtaW5kIl0 |
| #GOOG | Major Depression Checker | https://play.google.com/store/apps/details?id=net.ikko.majordepressionchecker&feature=search_result#?t=W251bGwsMSwxLDEsIm5ldC5pa2tvLm1ham9yZGVwcmVzc2lvbmNoZWNrZXIiXQ.. |
| #GOOG | Manifest Heal Relaxation | https://play.google.com/store/apps/details?id=com.mozinc.manifestheal&feature=search_result#?t=W251bGwsMSwxLDEsImNvbS5tb3ppbmMubWFuaWZlc3RoZWFsIl0 |
| #GOOG | Men and Depression | https://play.google.com/store/apps/details?id=com.swa.swamenanddepression&feature=search_result#?t=W251bGwsMSwxLDEsImNvbS5zd2Euc3dhbWVuYW5kZGVwcmVzc2lvbiJd |
| #GOOG | Mindfulness TS | https://play.google.com/store/apps/details?id=com.rep.MindFul&feature=search_result#?t=W251bGwsMSwxLDEsImNvbS5yZXAuTWluZEZ1bCJd |
| #GOOG | Mood Disorder Manual | https://play.google.com/store/apps/details?id=com.max.maxmooddisordermanual&feature=search_result#?t=W251bGwsMSwxLDEsImNvbS5tYXgubWF4bW9vZGRpc29yZGVybWFudWFsIl0. |

| MARKET | APP NAME | LINK |
|---|---|---|
| #GOOG | Mood Elevator & Support | https://play.google.com/store/apps/details?id=com.mobihypnos.soft.add69&feature=search_result#?t=W251bGwsMSwxLDEsImNvbS5tb2JpaHlwbm9zLnNvZnQuYWRkNjkiXQ |
| #GOOG | Mood Tracker By: CTHF | https://play.google.com/store/apps/details?id=com.greilly.moodtracker&feature=search_result#?t=W251bGwsMSwxLDEsImNvbS5ncmVpbGx5Lm1vb2R0cmFja2VyIl0. |
| #GOOG | Mood Tracker Depression | https://play.google.com/store/apps/details?id=org.radiantmonkeysoftware.moodtrackerdepression&feature=search_result#?t=W251bGwsMSwxLDEsIm9yZy5yYWRpYW50bW9ua2V5c29mdHdhcmUubW9vZHRyYWNrZXJkZXByZXNzaW9uIl0. |
| #GOOG | Nature Sounds Relax and Sleep | https://play.google.com/store/apps/details?id=com.zodinplex.naturesound&feature=search_result#?t=W251bGwsMSwxLDEsImNvbS56b2RpbnBsZXgubmF0dXJlc291bmQiXQ |
| #GOOG | NIH Depression Information | https://play.google.com/store/apps/details?id=com.incelligence.android.searchnavnihdpi&feature=search_result#?t=W251bGwsMSwxLDEsImNvbS5pbmNlbGxpZ2VuY2UuYW5kcm9pZC5zZWFyY2huYXZuaWhkcGkiXQ |
| #GOOG | Om Chanting | https://play.google.com/store/apps/details?id=com.xlratech.app.omchanting&feature=search_result#?t=W251bGwsMSwxLDEsImNvbS54bHJhdGVjaC5hcHAub21jaGFudGluZyJd |
| #GOOG | Operation Reach Out | https://play.google.com/store/apps/details?id=suicide.prevention.app&feature=search_result#?t=W251bGwsMSwxLDEsInN1aWNpZGUucHJldmVudGlvbi5hcHAiXQ |
| #GOOG | Overcome Anxiety | https://play.google.com/store/apps/details?id=com.bestourism.overcomeanxiety&feature=search_result#?t=W251bGwsMSwxLDEsImNvbS5iZXN0b3VyaXNtLm92ZXJjb21lYW54aWV0eSJd |
| #GOOG | Overcoming Depression | https://play.google.com/store/apps/details?id=com.koolappz.EP77707460001&feature=search_result#? |

| MARKET | APP NAME | LINK |
|---|---|---|
| | | t=W251bGwsMSwxLDEsImNvbS5rb29sYXBwei5FUDc3NzA3NDYwMDAxIl0 |
| #GOOG | Overcoming Depression Thing | https://play.google.com/store/apps/details?id=com.mobilenicheapps.overcomingdepressionthing&feature=search_result#?t=W251bGwsMSwxLDEsImNvbS5tb2JpbGVuaWNoZWFwcHMub3ZlcmNvbWluZ2RlcHJlc3Npb250aGluZyJd |
| #GOOG | Pain & Depression Ambiscience | https://play.google.com/store/apps/details?id=com.teslasoftware.android.ambiscience.pdr&feature=search_result#?t=W251bGwsMSwxLDEsImNvbS50ZXNsYXNvZnR3YXJlLmFuZHJvaWQuYW1iaXNjaWVuY2UucGRyIl0. |
| #GOOG | Positive Activity Jackpot | https://play.google.com/store/apps/details?id=t2.paj&feature=search_result#?t=W251bGwsMSwxLDEsInQyLnBhaiJd |
| #GOOG | Positive Thinking | https://play.google.com/store/apps/details?id=quotes.positivethinking&feature=search_result#?t=W251bGwsMSwxLDEsInF1b3Rlcy5wb3NpdGl2ZXRoaW5raW5nIl0 |
| #GOOG | Postnatal Depression- G. Lordi | https://play.google.com/store/apps/details?id=com.imobilize.postnataldepressiongl&feature=search_result#?t=W251bGwsMSwxLDEsImNvbS5pbW9iaWxpemUucG9zdG5hdGFsZGVwcmVzc2lvbmdsIl0. |
| #GOOG | PPD Gone! | https://play.google.com/store/apps/details?id=com.app_ppdgone.layout&feature=search_result#?t=W251bGwsMSwxLDEsImNvbS5hcHBfcHBkZ29uZS5sYXlvdXQiXQ |
| #GOOG | Prayers for Surv. Depression | https://play.google.com/store/apps/details?id=com.aldiko.android.oreilly.isbn9780819859761&feature=search_result#?t=W251bGwsMSwxLDEsImNvbS5hbGRpa28uYW5kcm9pZC5vcmVpbGx5LmlzYm45NzgwODE5ODU5NzYxIl0 |
| #GOOG | Psychological Tests | https://play.google.com/store/apps/details?id=com.prettyplanet.testsenglish&feature=search_result#?t=W251bGwsMSwxLDEsImNvbS5wcmV0dHlwbGFuZXQudGVzdHNlbmdsaXNoIl0 |
| #GOOG | Psychological Tests (Full) | https://play.google.com/store/apps/details?id=com.androidforwoman.psytestsengfull&feature=search_result#?t=W251bGwsMSwxLDEsImNvbS5hbmRyb2lkZm9yd29t |

| MARKET | APP NAME | LINK |
|---|---|---|
| | | YW4ucHN5dGVzdHNlbmdmdWxsIl0 |
| #GOOG | Qi Gond Medication and Relaxation | https://play.google.com/store/apps/details?id=com.excelatlife.motivation&feature=search_result#?t=W251bGwsMSwxLDEsImNvbS5leGNlbGF0bGlmZS5tb3RpdmF0aW9uIl0. |
| #GOOG | Refresh Your Brain Relaxation | https://play.google.com/store/apps/details?id=com.mozinc.refreshyourbrain&feature=search_result#?t=W251bGwsMSwxLDEsImNvbS5tb3ppbmMucmVmcmVzaHlvdXJicmFpbiJd |
| #GOOG | Relaxation Destination | https://play.google.com/store/apps/details?id=com.powerapps.relaxationdestination&feature=search_result#?t=W251bGwsMSwxLDEsImNvbS5wb3dlcmFwcHMucmVsYXhhdGlvbmRlc3RpbmF0aW9uIl0 |
| #GOOG | Showering Dream Relaxation | https://play.google.com/store/apps/details?id=com.mozinc.showeringdream&feature=search_result#?t=W251bGwsMSwxLDEsImNvbS5tb3ppbmMuc2hvd2VyaW5nZHJlYW0iXQ |
| #GOOG | Smiley Slinger | https://play.google.com/store/apps/details?id=com.dylan.slingshot&feature=search_result#?t=W251bGwsMSwxLDEsImNvbS5keWxhbi5zbGluZ3Nob3QiXQ |
| #GOOG | Speak Jesus Christ | https://play.google.com/store/apps/details?id=appinventor.ai_4nohype.iam&feature=search_result#?t=W251bGwsMSwxLDEsImFwcGludmVudG9yLmFpXzRub2h5cGUuaWFtIl0 |
| #GOOG | Stomping Out Depression | https://play.google.com/store/apps/details?id=com.bridgetree.android.tablet.stomping.out.depression&feature=search_result#?t=W251bGwsMSwxLDEsImNvbS5icmlkZ2V0cmVlLmFuZHJvaWQudGFibGV0LnN0b21waW5nLm91dC5kZXByZXNzaW9uIl0. |
| #GOOG | Stress - Anxiety - Depression | https://play.google.com/store/apps/details?id=com.andromo.dev117641.app125032&feature=search_result#?t=W251bGwsMSwxLDEsImNvbS5hbmRyb21vLmRldjExNzY0MS5hcHAxMjUwMzIiXQ.. |
| #GOOG | Stress Test and CBT Self-Help | https://play.google.com/store/apps/details?id=com.excelatlife.stress&feature=search_result#? |

| MARKET | APP NAME | LINK |
|---|---|---|
| | | t=W251bGwsMSwxLDEsImNvbS5leGNlbGF0bGlmZS5zdHJlc3MiXQ |
| #GOOG | Surviving Depression | https://play.google.com/store/apps/details?id=com.aldiko.android.oreilly.isbn9780819871954&feature=search_result#?t=W251bGwsMSwxLDEsImNvbS5hbGRpa28uYW5kcm9pZC5vcmVpbGx5LmlzYm45NzgwODE5ODcxOTU0Il0 |
| #GOOG | Surviving Depression | https://play.google.com/store/apps/details?id=org.paulin.sd&feature=search_result#?t=W251bGwsMSwxLDEsIm9yZy5wYXVsaW4uc2QiXQ.. |
| #GOOG | Symptoms of Depression | https://play.google.com/store/apps/details?id=com.wSymptomsOfDepression&feature=search_result#?t=W251bGwsMSwxLDEsImNvbS53U3ltcHRvbXNPZkRlcHJlc3Npb24iXQ |
| #GOOG | Teen Depression Connect | https://play.google.com/store/apps/details?id=com.alliancehealth.teendepressionconnect&feature=search_result#?t=W251bGwsMSwxLDEsImNvbS5hbGxpYW5jZWhlYWx0aC50ZWVuZGVwcmVzc2lvbmNvbm5lY3QiXQ.. |
| #GOOG | Teenage Depression | https://play.google.com/store/apps/details?id=com.koolappz.EP77900910001&feature=search_result#?t=W251bGwsMSwxLDEsImNvbS5rb29sYXBwei5FUDc3OTAwOTEwMDAxIl0 |
| #GOOG | Ten Tips to Ease Depression | https://play.google.com/store/apps/details?id=com.tendepressionreva&feature=search_result#?t=W251bGwsMSwxLDEsImNvbS50ZW5kZXByZXNzaW9ucmV2YSJd |
| #GOOG | The 72 Names of God | https://play.google.com/store/apps/details?id=com.kabbalah.the72names&feature=search_result#?t=W251bGwsMSwxLDEsImNvbS5rYWJiYWxhaC50aGU3Mm5hbWVzIl0 |
| #GOOG | The Art of Loving Yourself | https://play.google.com/store/apps/details?id=com.appmk.magazine.AOTINCZLYVUKCSPV&feature=search_result#?t=W251bGwsMSwxLDEsImNvbS5hcHBtay5tYWdhemluZS5BT1RJTkNaTFlWVUtDU1BWIl0 |
| #GOOG | The Depression Predictor | https://play.google.com/store/apps/details?id=com.thedoctorsays.depressionpredictor&feature=search_result#?t=W251bGwsMSwxLDEsImNvbS50aGVkb2N0b3JzYXlz |

| MARKET | APP NAME | LINK |
|---|---|---|
| | | LmRlcHJlc3Npb25wcmVkaWN0b3IiXQ |
| #GOOG | The Natural Depression Cures | https://play.google.com/store/apps/details?id=com.NaturalDepressionCures.AOTECEPPURCCMDTNA&feature=search_result#?t=W251bGwsMSwxLDEsImNvbS5OYXR1cmFsRGVwcmVzc2lvbkN1cmVzLkFPVEVDRVBQVVJDQ01EVE5BIl0 |
| #GOOG | Thunder Nap Sleep | https://play.google.com/store/apps/details?id=com.mozinc.thundernap&feature=search_result#?t=W251bGwsMSwxLDEsImNvbS5tb3ppbmMudGh1bmRlcm5hcCJd |
| #GOOG | Thunder Relief Relaxations | https://play.google.com/store/apps/details?id=com.mozinc.thunderrelief&feature=search_result#?t=W251bGwsMSwxLDEsImNvbS5tb3ppbmMudGh1bmRlcnJlbGllZiJd |
| #GOOG | Tips to Beat Depression | https://play.google.com/store/apps/details?id=com.BeatDepressionToday.book.AOTQRFWGFSYDRZKOF&feature=search_result#?t=W251bGwsMSwxLDEsImNvbS5CZWF0RGVwcmVzc2lvblRvZGF5LmJvb2suQU9UUVJGV0dGU1lEUlpLT0YiXQ.. |
| #GOOG | Treatment for Depression | https://play.google.com/store/apps/details?id=com.wTreatmentForDepression&feature=search_result#?t=W251bGwsMSwxLDEsImNvbS53VHJlYXRtZW50Rm9yRGVwcmVzc2lvbiJd |
| #GOOG | Truth About BiPolar Disorder | https://play.google.com/store/apps/details?id=com.TruthAboutBiPolarDisorder.book.AOTEOFGQFVJAQSAYVL&feature=search_result#?t=W251bGwsMSwxLDEsImNvbS5UcnV0aEFib3V0QmlQb2xhckRpc29yZGVyLmJvb2suQU9URU9GR1FGVkpBUVNBWVZMIl0 |
| #GOOG | Vanitas | https://play.google.com/store/apps/details?id=com.taleoftales.Vanitas&feature=search_result#?t=W251bGwsMSwxLDEsImNvbS50YWxlb2Z0YWxlcy5WYW5pdGFzIl0 |
| #GOOG | Weed Strains 3D+ Join Together | https://play.google.com/store/apps/details?id=com.alphakua.jointogether&feature=search_result#?t=W251bGwsMSwxLDEsImNvbS5hbHBoYWt1YS5qb2ludG9nZXRoZXIiXQ.. |
| #GOOG | WhatsMyM3 | https://play.google.com/store/apps/details?id=com.mymoodmonitor.whatsmym3&feature=search_r |

| MARKET | APP NAME | LINK |
|---|---|---|
| | | esult#?<br>t=W251bGwsMSwxLDEsImNvbS5teW1vb2Rtb25pdG9yLndoYXRzbXltMyJd |
| #GOOG | Words that Changed Lives | https://play.google.com/store/apps/details?<br>id=com.highonsms.famouspeoplequotes&feature=search_result#?<br>t=W251bGwsMSwxLDEsImNvbS5oaWdob25zbXMuZmFtb3VzcGVvcGxlcXVvdGVzIl0 |
| #NOKIA | Business Motivation | http://store.ovi.com/content/327244?<br>clickSource=search&pos=4 |
| #NOKIA | Health Tips | http://store.ovi.com/content/351614?<br>clickSource=search&pos=3 |
| #NOKIA | How Depressed Are You? | http://store.ovi.com/content/298105?<br>clickSource=search&pos=5 |
| #NOKIA | Italk to God - Message, NRSV, NASB | http://store.ovi.com/content/23030?<br>clickSource=search&pos=5 |
| #NOKIA | Mental Health for Muslims | http://store.ovi.com/content/44607?<br>clickSource=search&pos=4 |
| #NOKIA | MoodJournal | http://store.ovi.com/content/18360?<br>clickSource=search&pos=10 |
| #WINDOWS | Barometer | http://www.windowsphone.com/en-ca/store/app/barometer/7b54e1e0-5104-4413-ad29-4d2262c77474 |
| #WINDOWS | Depression Aid | http://www.windowsphone.com/en-ca/store/app/depression-aid/3b9ba662-a0ad-46f4-9568-671e04738c3c |
| #WINDOWS | Depression Test | http://www.windowsphone.com/en-ca/store/app/depression-test/82b5ad73-1ea7-4d14-ba57-8bf2db6879bb |
| #WINDOWS | Depression Test (Quick) | http://www.windowsphone.com/en-ca/store/app/depression-test-quick/89bc4d44-05ef-45ab-a853-df686d0cdc57 |
| #WINDOWS | Heal Depression | http://www.windowsphone.com/en-ca/store/app/heal-depression/6dd07478-fd44-40ab-a819-34a3c428b2cf |
| #WINDOWS | Hynogear | http://www.windowsphone.com/en-ca/store/app/hg/dacd32a5-a687-4a05-a3d7-054550ba47f2 |
| #WINDOWS | Light at the End fo the Tunnel | http://www.windowsphone.com/en-ca/store/app/light-at-the-end-of-the-tunnel/3ce90f49-4f4d-40e0-9420-71f6d83604c7 |
| #WINDOW | Mood Tracker | http://www.windowsphone.com/en- |

| MARKET | APP NAME | LINK |
|---|---|---|
| S | | ca/store/app/moodtracker/98bbaba0-6b1c-4eb7-a512-d2b387d89d03 |
| #WINDOWS | Mood Tracker (Full) | http://www.windowsphone.com/en-ca/store/app/moodtracker/e7159641-69bb-4034-af96-dca3714386df |
| #WINDOWS | The Yellow Wallpaper by Listen and Live Audio | http://www.windowsphone.com/en-ca/store/app/the-yellow-wallpaper-by-listen-live-audio/88404b57-5c46-42b2-884a-1b5bb576bef8 |
| #WINDOWS | What Now | http://www.windowsphone.com/en-ca/store/app/what-now/1753b84e-ed52-4253-a936-cde0c4f71312 |
